# ICE-CAP: ice crystal engineering for cryogel architecture programming. Modulation of material properties with ice-binding polymers

**DOI:** 10.1039/d6mh01950a

**Published:** 2026-10-22

**Authors:** Cigdem Buse Oral, Libby J. Marshall, Dave J. Adams, Matthew I. Gibson

**Affiliations:** a Department of Chemistry, University of Manchester Oxford Road Manchester M13 9PL UK matt.gibson@manchester.ac.uk; b Manchester Institute of Biotechnology, University of Manchester 131 Princess Street Manchester M1 7DN UK; c School of Chemistry, University of Glasgow Glasgow G12 8QQ UK

## Abstract

Macroporous cryogels are formed by polymerising monomers around ice crystal templates, with water as the only waste product, making them appealing as tuneable and sustainable scaffolds. Cryogels have found diverse applications spanning pollutant removal, vaccine delivery, tissue culture and purification. However, an unmet challenge remains: ice crystal templates are intrinsically dynamic and evolve during polymerisation, leading to heterogeneity and complicating reproducibility. Here we overcome this challenge using ice-binding protein-inspired polymers that pre-program the ice crystal template size within cryogels through selective ice crystal surface binding. This ice-crystal programming is shown to fine-tune the rheological and mechanical properties of the resulting materials, without any change in the monomer composition or extensive optimization of reaction conditions, unlike conventional methods. This approach unlocks wider applications of cryogels, exploiting biomimetic solutions to tune the crucial ice crystal template.

## Abstract 2

A bio-inspired strategy is introduced to programme ice crystal templates growth during cryogel formation, enabling precise control over material properties without changing the polymer components.

## Graphical abstract

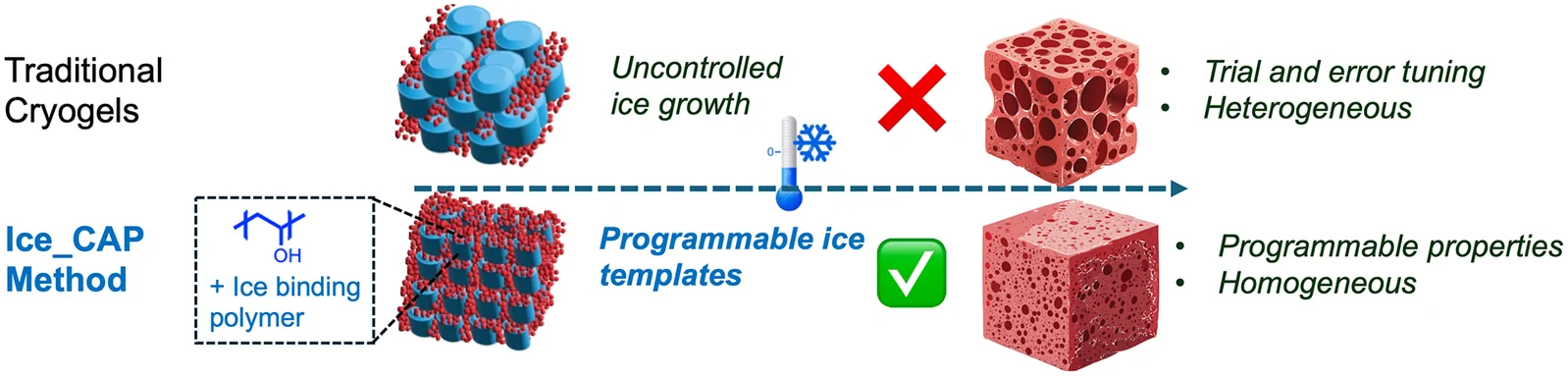

**New concepts**

To prepare porous materials, templates are required, such as inorganic colloids. Ice is a highly desirable template, as it is low-cost, sustainable and non-toxic, and can be easily removed by melting or lyophilization. The fundamental problem, however, with using ice is that unlike inorganic templates, it is dynamic: its size and shape evolve during cryogel formation. Here we make a conceptual advance by using biomimetic polymers that bind ice crystals to pre-program their size, and hence the porosity and material properties of the resultant cryogels. We show that the distribution and size of pores are controllable by addition of as little as 0.1 wt% of ice-binding polymers. This precision approach can replace the trial/error and iterative formulation that is normally required to tune cryogels. By programming the ice crystal template, we show that the mechanical properties and chromatographic separation properties of the materials are fully tuneable without changing the cryogel scaffolding material itself. Hence, this is a broadly applicable, scalable and versatile porous material tool.

## Introduction

Porous polymeric materials are used in a wide range of applications, due to their interconnected networks, large surface areas, easily adjustable pore sizes and high porosity. Porous materials are ubiquitous in the modern world, finding applications in various fields including (non-exclusively) gas adsorption,^1,2^ catalysis,^3,4^ gas storage,^5,6^ drug delivery^7,8^ and tissue engineering.^9,10^

To generate porosity, a wide range of methods are used, such as additive manufacturing, selective degradation, gas foaming and phase separation. A particularly powerful method is based on removable templates, whereby the polymer forms around the template, recapitulating the size/shape of the templates. These templates can include colloids, emulsions, high-internal-phase emulsions (HIPEs),^11^ micelles,^12^ block copolymer self-assemblies,^13,14^ sacrificial polymer or gel networks,^15–17^ molecular porogens,^18,19^ and gas-foaming agents.^20,21^

Cryogels are an interesting class of porous materials in which the templates are ice crystals, which can be removed post-synthesis by washing or lyophilization. In a typical cryogelation, the monomer solution is frozen, and as ice forms a pure phase, the monomer concentrates around the templates, which locally increases the monomer concentration, giving rise to the interconnected microporous polymer network. Ice is an appealing template for sustainable processes and for forming large macropores (typically 10–500 microns). Cryogels have been explored in a wide range of applications, including tissue engineering scaffolds, injectable drug-delivery matrices, cell carriers and bioreactors, wound dressings, biosensors, and adsorbents for environmental remediation, and have been prepared from diverse material classes.^22–24^ For example, poly(acrylamide) cryogels prepared by free-radical polymerisation under frozen conditions have been used as cell-delivery matrices and tissue scaffolds.^25,26^ Besides radical polymerisation, ionically crosslinked alginate cryogels have been developed as injectable and shape-recoverable scaffolds.^27^ Cryogels made from natural polymers such as gelatin and chitosan, formed through either physical or chemical crosslinking, have also been utilised in wound healing and regenerative medicine.^28,29^ Composite and hybrid cryogels containing nanoparticles or bioactive components, such as hyaluronic acid cryogels containing calcium peroxide, have also been reported for biosensing, environmental remediation and tissue engineering applications.^30,31^

Despite their broad applicability, cryogels face several challenges, particularly the need to fine-tune the formation (nucleation) and growth of ice crystals, which under standard conditions results in a heterogeneous size distribution. Several established parameters can be used to modulate the ice crystal template: the freezing temperature,^32,33^ the precursor concentration,^34^ the freezing rate,^35,36^ the ice nucleation temperature^37^ and directional freezing,^38^ imposing anisotropic, aligned pore channels. Additives such as the cryoprotectant dimethyl sulfoxide (DMSO) have been used to regulate ice crystal formation through freezing-point depression, allowing adjustment of the pore size and stiffness of the resulting cryogels.^39^ These methods are effective but share a common limitation: they are intrinsically coupled to other properties. For example, increasing or decreasing the temperature changes the cryoconcentration, increasing the precursor solute concentration changes the crosslink density and changing the freezing direction results in anisotropy. These variables act on the ice template only indirectly and depend on one another. Pore size cannot be adjusted without simultaneously changing the composition, crosslink density, or mechanical properties, and the desired pore size is typically achieved only through extensive trial and error.

In contrast to the above materials-science approach, Nature has evolved ice binding proteins^40^ (IBPs), which can modulate both the formation^41,42^ (nucleation) and growth of ice crystals,^43,44^ enabling extremophiles to survive in sub-zero habitats through ice crystal engineering. To unlock translation and tunability of the IBPs, synthetic (macro)molecular mimics^45–47^ of IBPs have emerged, including poly(vinyl alcohol),^48,49^ graphenics,^50,51^ polypeptides^52,53^ and small molecules.^54–57^

Considering the above properties of ice binding proteins, we hypothesised that synthetically scalable and tuneable ice-binding polymers could be used to engineer and pre-program the size and distribution of the ice crystal templates to unlock a new generation of tuneable cryogels, making use of water/ice as a fully sustainable porogen (Fig. 1). Controlling ice crystal size and morphology has been explored to tune the structure of inorganic materials (particularly by directional freezing)^58,59^ but, to the best of our knowledge, there is only a single report on the use of a type III antifreeze protein in an alginate gel to support the culture of chondrocytes,^60^ which, however, did not explore the tunability nor link quantitative ice growth assays to the macroscopic structure of the resulting materials.

**Fig. 1:**
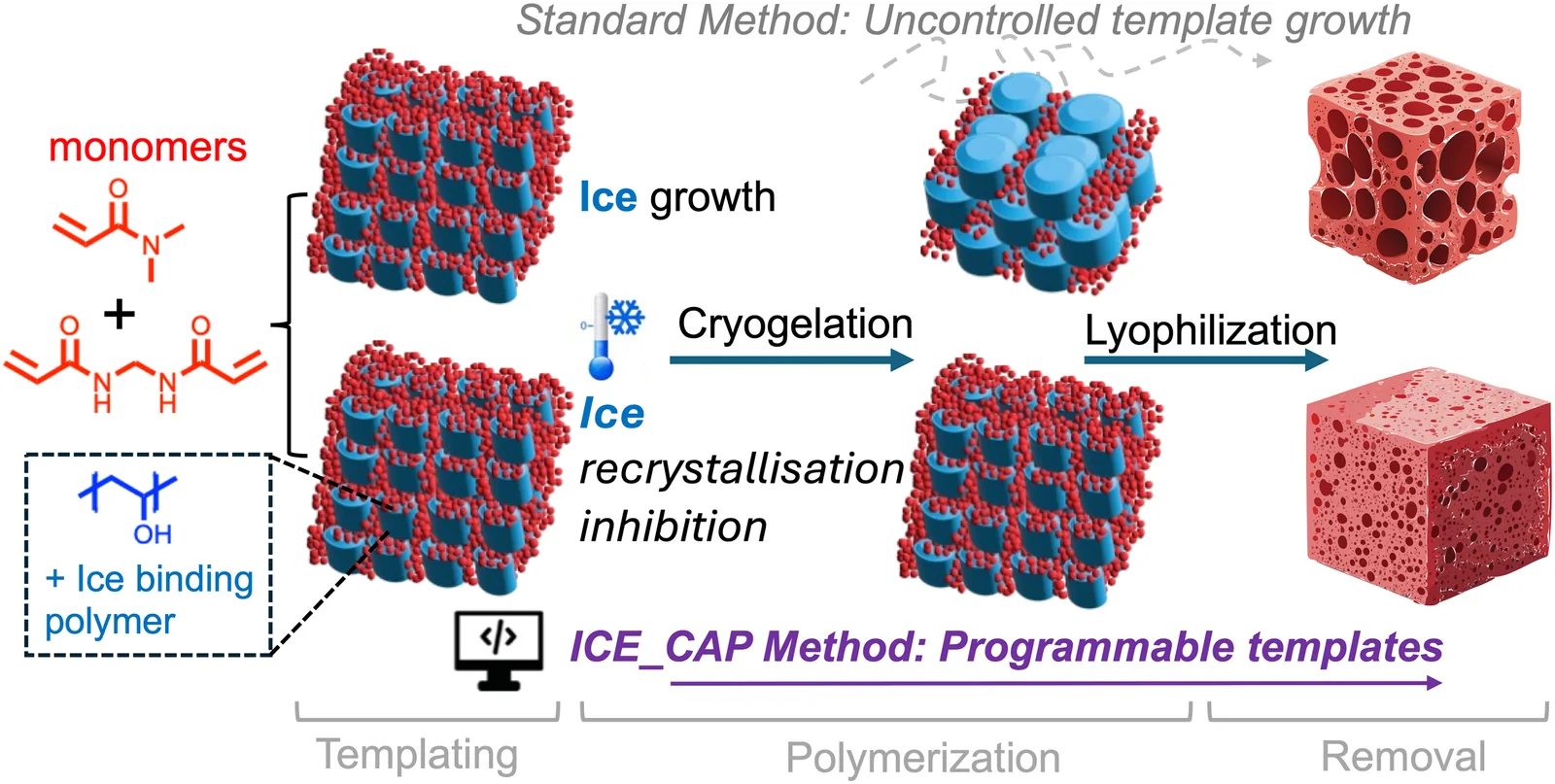
Ice crystal engineering for cryogel architecture programming (ICE-CAP) process. Monomers are templated around ice crystals at sub-zero temperatures with or without ice binding polymers (poly(vinyl alcohol), PVA). During cryogelation, the ice binding polymer concentration programs the size of the ice crystals. After removal of ice, the microporous gel porosity is determined using the programmed ice crystal size.

Here we demonstrate that the synthetic ice binding polymer, PVA, can be used to control the size and growth rate of ice crystals in a monomer solution under these conditions and that the resultant gels have pore sizes and distributions correlating to these ice crystals (Fig. 1). It is crucial to note that the gel is not made from PVA, which is a minor component, and this is distinct from well-known PVA-only cryogels. Rheological and mechanical testing demonstrate that ice-crystal tuning unlocks pore engineering to impart control over the macroscopic properties of the gels, without the need to change the composition or polymerisation temperature. Model chromatographic studies highlight that improved separations can be achieved by tuning pore size.

## Results and discussion

To obtain cryogels, the monomer dimethylacrylamide (DMAA) was chosen as it is uncharged, highly water soluble and has been used previously, to allow critical comparisons.^61^ To tune ice crystal (and hence pore) size, PVA was chosen as the biomimetic ice recrystallisation inhibitor, as it is synthetically scalable and low-cost and cannot denature (even at extreme pH^62,63^), ensuring that activity is retained across a broad spectrum of solution conditions, which cannot be guaranteed with proteins. The mechanism of action of PVA to engage ice has been widely studied.^48,49,64^

PEG was chosen as the negative control as it has no IRI (ice recrystallisation inhibition) activity and is isomeric to PVA, to ensure observations are not simply due to a high molecular weight dopant. The presence of solutes (such as salts, acid/bases and the monomers) impacts the ice growth process,^62,65^ so we first investigated how PVA or PEG (0 to 10 mg mL^−1^) in DMAA monomer solutions impacts ice growth using the splat assay at a range of sub-zero temperatures.^45,66^ Briefly, a polynucleated wafer of ice crystals is formed, which is then annealed at the given temperature, and their size is measured after 30 minutes: smaller ice crystals indicate more activity. Fig. 2 shows that at −5 °C and −10 °C, there was significant ice crystal growth, which is indicative of the polymers having limited IRI under these temperature and solution conditions. This observation is crucial, as the IRI (splat) assays are typically conducted in this temperature range and show how the complex monomer solution does not correlate with standard saline conditions.^45,66^ In contrast, wafers annealed at −20 and −24 °C revealed a specific dose-dependent response between PVA concentration and reduced ice crystal size. These first experiments are crucial, revealing that the IRI of PVA in monomer solution is less than that in standard saline^45,48,67^ but complete inhibition is achieved with 5–10 mg mL^−1^. This also justifies the use of PVA rather than IBPs, as the cost/availability of IBPs, if higher concentrations are needed, is a limiting factor and the potential for cross-reactivity (*e.g.*, Michael addition to monomers by lysine/cystine residues) is eliminated by using PVA. Furthermore, −20 °C is also readily achievable in a standard laboratory freezer, compared to higher sub-zero temperatures which require dedicated facilities. Differential scanning calorimetry (Fig. S6) showed a decrease in total frozen fraction upon addition of PVA, which correlates with the above.

**Fig. 2:**
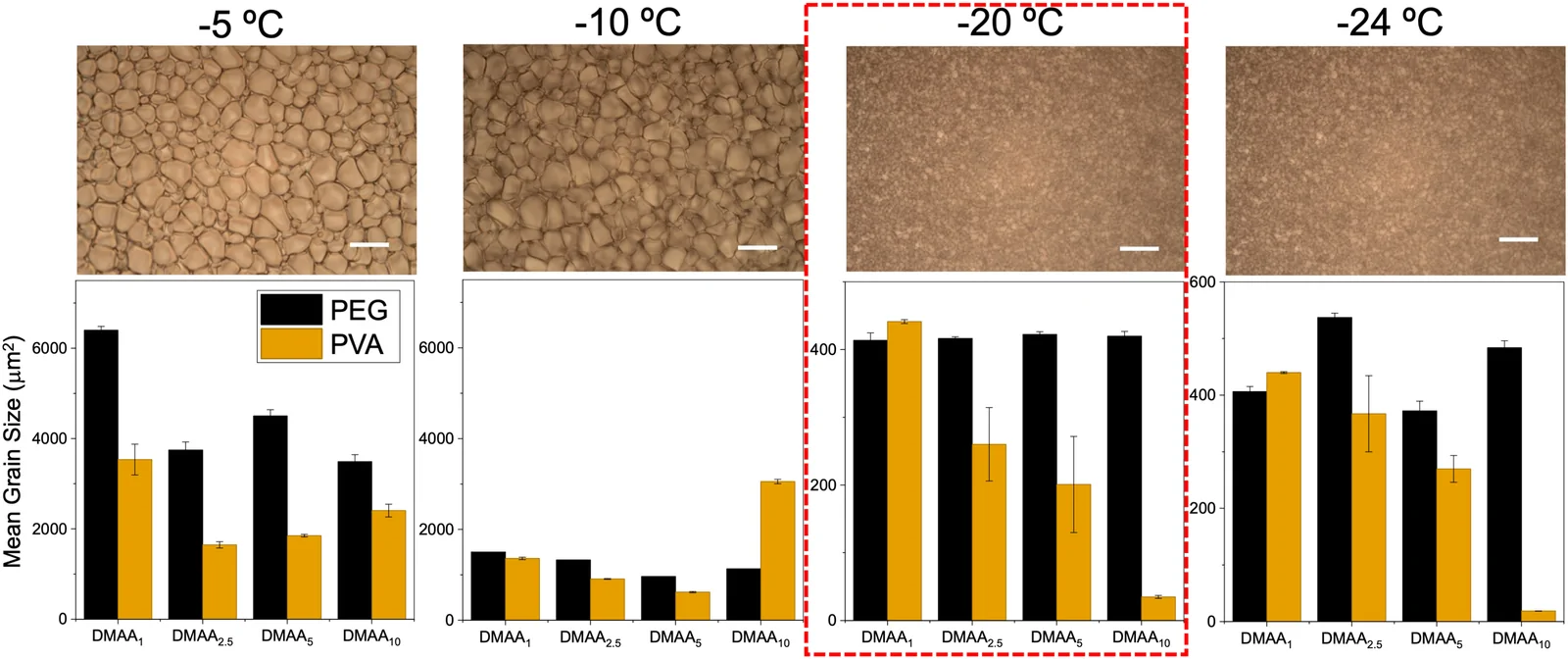
Impact of PVA and PEG on ice recrystallisation in DMAA monomer solutions. In each column, cryomicrographs show ice crystals after 30 minutes of annealing at the indicated temperatures with 2.5 mg mL^−1^ PVA. Bar charts show the mean ice crystal grain size after annealing at the indicated temperature with PVA/PEG added. DMAA*x*, where *x* = [polymer] in mg mL^−1^. *n* = 3. The red box highlights that −20 °C was identified as the optimum temperature for ice growth control in DMAA solutions. Scale bar = 100 µm. The mean ice grain size was quantified from three independent images per condition (*n* = 3) and reported as mean ± S.D.

Guided by the above, macroporous cryogels were prepared using DMAA (0.5 M) with *N*,*N*′-methylenebis(acrylamide) (MBAA) as the crosslinker and ammonium persulfate (APS) with *N*,*N*,*N*′,*N*′-tetramethyl ethylenediamine (TEMED) as the redox initiator system and allowed to polymerise at a range of temperatures with different amounts of PVA. The polymerisation proceeded rapidly and, in all cases, led to free-standing gels which could be reswollen and dried multiple times (Fig. 3A). The ice templates were removed by freeze-drying and imaged using SEM (scanning electron microscopy) to visualize the internal structure of the gels and determine pore size. Cryogels produced at −5 °C had pore sizes (from SEM) with a broad distribution centred around 100 µm and in line with the above splat assay data (Fig. 2), and there was limited impact of the PVA addition. In sharp contrast, at −20 °C the pore size decreased with increasing PVA and the size distribution was narrower. At −20 °C, the cryogels appear to form aligned, channel-like pores. Because these channels run through the material, the fracture plane cuts some of them lengthwise, so they appear as elongated features in the SEM images even though their true width is small. These are not a distinct population of larger pores; to avoid this artefact, we measured pore size throughout as the equivalent cross-sectional diameter. We would also note that higher concentrations of PVA can lead to ice-crystal shaping (due to selective crystal face binding), which might give rise to anisotropic templates, but this was avoided at lower concentrations, which were sufficient to still limit recrystallisation. These data provide clear evidence that by modulating IRI activity, the internal structure of the crosslinked gels can be modulated and tuned. This is far simpler and more versatile than standard methods for adjusting the monomer concentration,^68^ creating a second network^69^ or changing the monomer and/or temperature.^70^ Confocal fluorescence microscopy was also used to image the gels, using fluorescein for contrast, revealing a homogeneous structure when PVA was used to program ice growth.

**Fig. 3:**
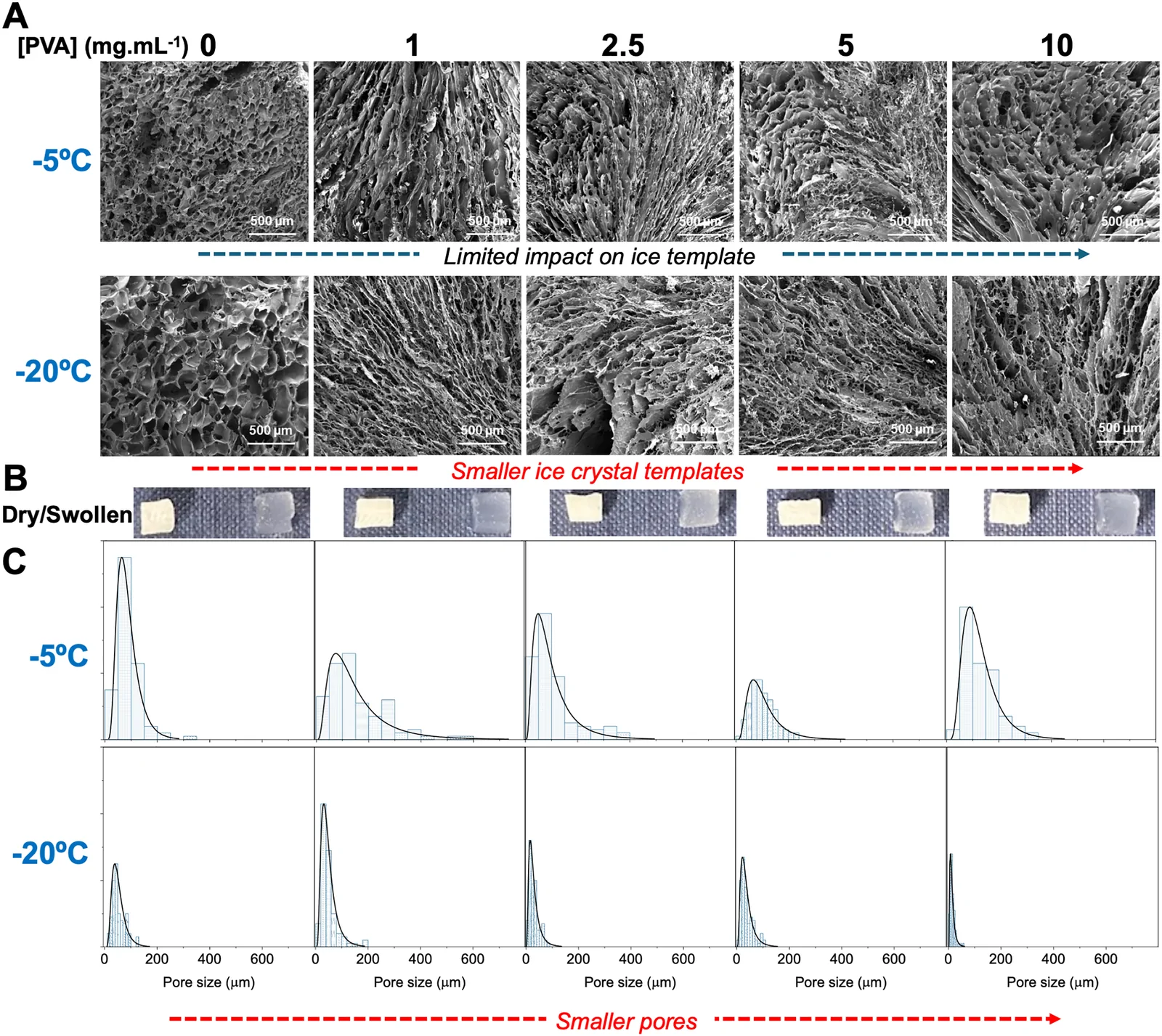
Macroporous cryogel pore analysis post-freeze drying. (A) Scanning electron microscopy images of DMAA cryogels prepared at the indicated temperature, as a function of [PVA]; (B) photographs of dry/swollen cryogels; (C) pore size distribution (from SEM) of DMAA cryogels prepared at the indicated temperature, as a function of [PVA] (indicated at the top of the figure). Pore sizes were measured as equivalent circular diameters using ImageJ (three images per condition and 100 pores per image, reporting true pore width independently of channel orientation).

To investigate if this precise tuning of the pore size and connectivity impacted the bulk material properties of the gels, oscillatory rheology was conducted and the elastic modulus was calculated (Fig. 4). The gels prepared at −5 °C showed small changes from ∼200 to 400 Pa upon addition of PVA, in line with the porosity and ice growth data showing that at this temperature, PVA did not strongly impact ice growth and hence the elastic modulus was not impacted. In contrast, for the gels prepared at −20 °C, the elastic modulus was found to be highly tunable over a range of 400 Pa, with 5 mg mL^−1^ PVA giving the highest values. Mechanical testing was also employed showing that the fracture stress increased when 5 mg mL^−1^ PVA was added, but the fracture strain did not change, showing that pore size tuning can alter the stiffness of materials. As with the ice growth assays, we saw plateaus, or decreases, in mechanical properties when 10 mg mL^−1^ PVA was used. To establish the origin of this behaviour, we compared the true monomer concentration (*C*_true_) by DSC in the unfrozen phase with pore size and elastic modulus across the full PVA series (Table S1). *C*_true_ and the elastic modulus are inversely correlated at every concentration; in particular, the stiffest gel (5 mg mL^−1^ PVA) has the lowest *C*_true_ of the series, ruling out local reactant concentration as the primary determinant of stiffness and identifying pore architecture as the main driver of the mechanical properties. Turbidity and dynamic light scattering measurements of the precursor solutions (Fig. S11 and S12) further exclude bulk PVA aggregation or precipitation as a contributing factor. The modulus decrease specific to 10 mg mL^−1^ coincides with a significant decrease in the melting temperature (*T*_m_) of the unfrozen phase (relative to the near-constant *C*_true_ over this interval), consistent with PVA's established behaviours,^71^ and with its known freeze-induced self-association,^72^ either of which could locally disrupt the network and impose a softening effect on the pore-driven stiffening. This softening effect explains the decreased modulus at higher PVA concentrations and identifies 5 mg mL^−1^ as the optimum condition for our system. We obtained similar results for HEMA (hydroxyethyl methacrylate) cryogels, where PVA tunes pore size, and included them in the SI (Fig. S14). Overall, these data show that pore-size engineering impacts the macro-scale properties, without needing to tune the monomer concentration or cross-linkers, and directly links the biophysical ice growth assays to the final material properties, which will be essential for future optimization and deployment across a range of monomers and polymers.

**Fig. 4:**
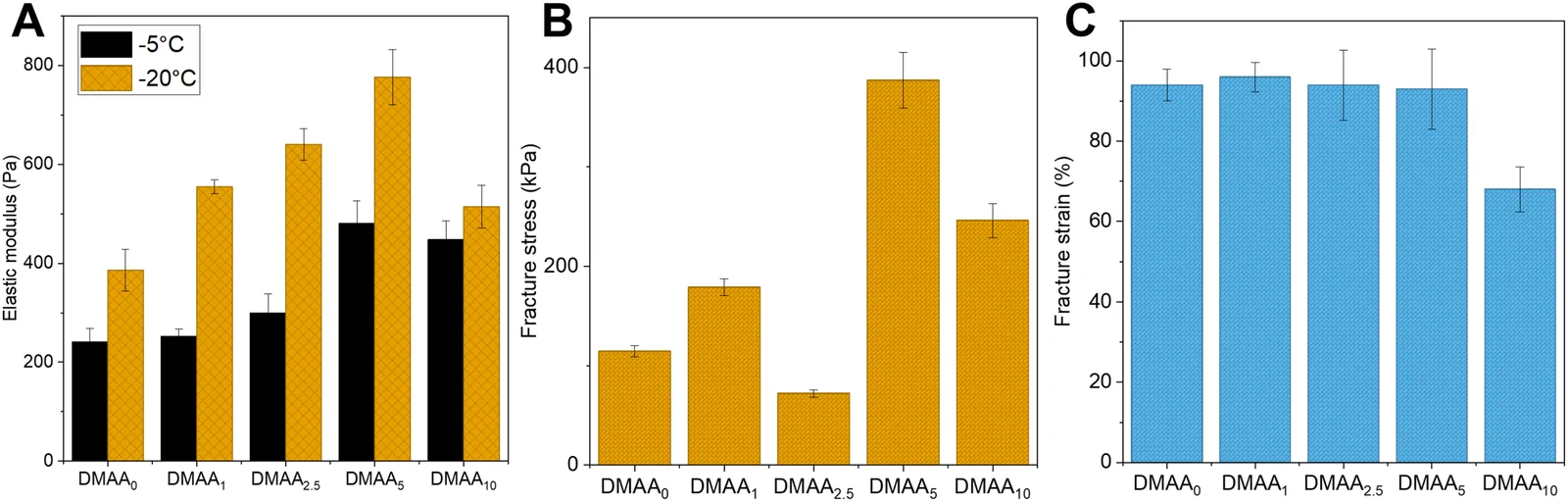
Physical properties of cryogels (in the hydrated state). (A) Elastic modulus as a function of the temperature at which the gels were prepared, as determined by oscillatory rheology. (B) Fracture stress and (C) fracture strain from mechanical analysis, as a function of [PVA] (and hence pore size) of the prepared cryogels.

Swelling measurements were also performed to assess whether the observed trends in bulk properties result from the ice-templated pore structure or changes in the polymer network (Fig. S3–S5). The gel fraction (*W*_g_) remained close to unity at all preparation temperatures, suggesting that network formation was efficient and that loss of uncrosslinked polymer during swelling was minimal (Fig. S3B). For cryogels prepared at −5 °C, both equilibrium water content (EWC, Fig. S3A) and porosity (*P*, Fig. S4B) changed only slightly with PVA addition, which is in line with the weak effect of PVA on ice growth and rheological properties at this temperature. By contrast, cryogels prepared at −20 °C showed lower EWC and reduced pore volume (*V*_p_, Fig. S4A) with increasing PVA concentration, consistent with smaller pore sizes and higher elastic moduli measured by rheology. Overall, the swelling data support the conclusion that the observed differences in bulk material behaviour arise from pore-size programming rather than from variations in gel integrity or crosslink density.

One application of porous media is the absorption or removal of pollutants and they also serve as the stationary phases for chromatographic separation. To investigate the impact of the tuneable porosity further, gels were prepared directly within syringes to test how solutes flow through these, as a model separation application. Methylene blue (20 µM) was applied to the swollen gels and the breakthrough time (the time required for the solute to pass through) was measured (Fig. 5). There was a clear trend that the gels prepared with 5 mg mL^−1^ PVA (smallest ice template) retained the dye for a longer duration compared to the native cryogels (larger template), which confirms that engineering smaller pore sizes leads to greater retention times and also that intermediate retention can be achieved by selecting lower PVA concentrations. In all cases, quantitative recovery of the dye was achieved. As a larger (∼27 kDa) cargo, recombinant mCherry (red fluorescent protein) was also passed through the columns, and in this case, the impact of porosity was very significant: the largest pores (no PVA) eluted mCherry in just 5 seconds, whereas the smallest pores (5 mg mL^−1^ PVA) retained the protein for 170 seconds. An additional experiment showed that separation of a mixture of these two solutes could be controlled through pore-size tuning (SI, Fig. S13). These observations demonstrate the power of this simple and tuneable method, which in principle can be used to separate particles based on their size, as well as interaction chromatography if functional monomers were employed. To achieve this using conventional approaches, a long optimization campaign involving monomer selection, cross-link density and freezing temperature would be required, which we accelerate.

**Fig. 5:**
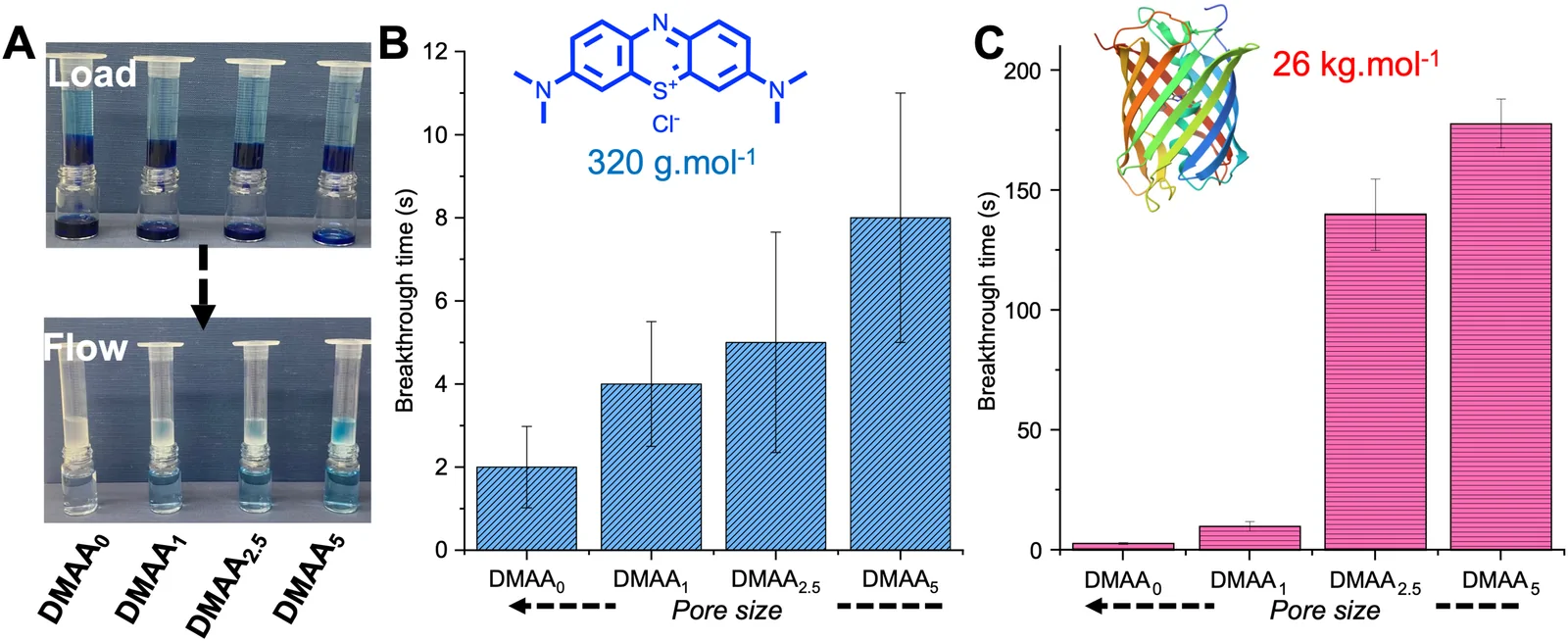
Chromatographic demonstration of the impact of pore-size programming by measuring the breakthrough time of different sized probes. (A) Photographs of cryogels (inside syringe) and flow of methylene blue; quantification of (B) methylene blue and (C) mCherry, with breakthrough time as a function of [PVA] and pore size. *n* = 5. DMAA*x* indicates the PVA concentration (mg mL^−1^) used to prepare the cryogel.

## Conclusions

Here we have introduced ICE-CAP: ice crystal engineering for cryogel architecture programming, whereby the structure, and hence function, of cryogels can be fine-tuned and optimized by the use of ice-binding polymers to program the size and growth rates of the ice templates. This approach is significantly simpler and more tuneable than the standard iteration of reaction conditions such as monomer concentrations or freezing rates. It was first shown that poly(vinyl alcohol), a uniquely active ice-recrystallisation inhibitor (IRI), retains its IRI activity in complex monomer mixtures even at low concentrations. Compared to PEG controls, PVA allowed control of the ice crystal size, with −20 °C (a convenient temperature for conventional freezers) being optimal. The resulting microporous cryogels (after ice removal) were imaged by SEM and confocal microscopy, which revealed pore sizes corresponding to the ice templates, with increased PVA concentrations (smaller ice crystals) resulting in smaller pores than those obtained without PVA. Rheological and mechanical testing analyses showed that the elastic modulus correlated with the pore size (and hence the PVA concentration) and that cryogels engineered with smaller ice templates exhibited increased fracture stress. Model chromatographic separation studies using the cryogels as the solid phase showed that the retention time of dyes and proteins was fully tuneable and controllable by the size of the ice crystal template. Taken together, this evidence supports the use of ice-crystal engineering as a sustainable solution to advanced porous materials in which the properties are fully controlled by the templates, which are in turn controlled by the nature of the ice binding additives. Future studies will include exploration of biomedical applications as well as separations, while also expanding the range of pore sizes.

## Experimental section

Detailed experimental procedures and further characterization of all materials are included in the SI.

## Conflicts of interest

There are no conflicts to declare.

## Supplementary Material

MH-OLF-D6MH01950A-s001

## Data Availability

The data supporting this article have been included as part of the supplementary information (SI). Supplementary information is available. See DOI: https://doi.org/10.1039/d6mh01950a.
