# Supplementary material for: ICE-CAP: ice crystal engineering for cryogel architecture programming. Modulation of material properties with ice-binding polymers
Source: Mater Horiz. 2026 Oct 22. Online ahead of print. doi: 10.1039/d6mh01950a (PMC13688315; doi:10.1039/d6mh01950a)
Supplement: Supplementary file 1 [file MH-OLF-D6MH01950A-s001.pdf]

Supplementary Information (SI) for Materials Horizons.
This journal is © The Royal Society of Chemistry

Supporting Information for

ICE-CAP: Ice Crystal Engineering for Cryogel Architecture Programming. Modulation of Material Properties with Ice-Binding Polymers.

Cigdem Buse Oral,[a] Libby J. Marshall,[c] Dave J. Adams[c] and Matthew I. Gibson[a,b]*

[a] Department of Chemistry, University of Manchester, Oxford Road, Manchester, M13 9PL, UK

[b] Manchester Institute of Biotechnology, University of Manchester, 131 Princess Street, Manchester, M1 7DN, UK

[c] School of Chemistry, University of Glasgow, Glasgow, G12 8QQ, UK

E-mail: matt.gibson@manchester.ac.uk

Experimental Section

Materials

N, N - dimethylacrylamide (DMAA, Sigma Aldrich, ≥99%), poly (vinyl alcohol) (PVA, Sigma Aldrich, M_w: 9000-10000, 80% hydrolysed), *N, N'*-methylene bis(acrylamide) (BAAm, Sigma Aldrich, ≥99%), ammonium persulfate (APS, Sigma Aldrich, ≥98%), *N, N, N', N'* – tetramethyl ethylenediamine (TEMED), PanReac AppliChem, ≥99%), acetone (EMD Millipore, ACS reagent, d= 0.784 g.mL^{-1}, ≥99.5%), sodium chloride (NaCl, extra pure, SLR, Fisher Chemical), poly(ethylene glycol) (PEG, Sigma Aldrich, M_n: 8000), methylene blue hydrate (MB, Thermo Fisher Scientific, ≥96%) and fluorescein isothiocyanate (FTIC, FluoroChem, ≥95%). All chemicals are used as received without further purification. mCherry was produced recombinantly in *Escherichia coli* BL21(DE3) and purified following previously reported methods [1].

Preparation of Cryogels

DMAA cryogels were prepared according to an established protocol, with modifications [2]. Unless otherwise stated, the concentrations of DMAA, BAAm, APS and TEMED were fixed at 50 mg. mL^{-1} (0.50 M), 0.97 mg. mL^{-1} (6.40 mM), 1.0 mg. mL^{-1} (4.40 mM) and 3.40 mg. mL^{-1} (33.40 mM), respectively. The molar ratio of BAAm to DMAA was kept at 1:80 for all samples. PVA concentration was changed from 0 to 10 mg. mL^{-1}.

To prepare a DMAA cryogel without PVA ($DMAA_0$) with a final volume of 1.0 mL, 941 µL of ultrapure water (σ = 18.2 MΩ.cm) was added to a glass vial placed in an ice bath, followed by the addition of 52 µL of DMAA monomer under gentle stirring. BAAm crosslinker (9.7 mg) was then added and allowed to dissolve completely, yielding a clear solution. The reaction solution was purged with nitrogen for 5 min to remove reactive oxygen species that could hinder free-radical polymerisation. Polymerisation was initiated by adding APS (1 mg) and TEMED (5 µL), then stirring for 1 min. Then, the reaction solution was immediately transferred into plastic syringes with a 4.6, 14.5- and 20-mm diameter and briefly immersed in liquid nitrogen. Syringes containing DMAA cryogel solutions were placed at -5, - 10, -20 and -24 ºC for 24 h. After polymerisation, the cryogels were thawed at room temperature and removed from the syringes. Dry cryogels were obtained using a freeze dryer (Chris-Alpha 1-4 LSC basic) at 1 mbar and -55 ºC for 72 h. DMAA cryogels containing PVA were prepared using the same procedure by incorporating PVA into the reaction solution at concentrations of 1, 2.5, 5,

and 10 mg mL^{-1}, denoted as $DMAA_1$, $DMAA_{2.5}$, $DMAA_5$, and $DMAA_{10}$, respectively. PVA was introduced using a 100 mg mL^{-1} aqueous stock solution, prepared by dissolving PVA in ultrapure water at 90 ºC until fully clear, then cooling to room temperature before use. For a final 1 mL volume of solution, 10, 25, 50, and 100 µL of PVA stock solution were added to obtain final PVA concentrations of 1, 2.5, 5, and 10 mg mL^{-1}, respectively, with the added volume compensated by reducing the water volume accordingly.

Characterisation of Cryogels

Unless otherwise stated, all measurements were performed on at least five samples.

Splat Ice Recrystallisation Inhibition (IRI) Assay

Splat ice recrystallisation inhibition (IRI) assays were performed as previously described [3]. Briefly, 10 µL of DMAA precursor solutions were dropped from a height of 1.40 m onto a chilled glass coverslip, which was placed on a thin aluminium block pre-cooled to -78 °C. Upon splatting solutions onto the coverslip, a wafer approximately 10 mm in diameter and 10 µm in thickness formed instantaneously. The glass coverslip was subsequently transferred onto the Linkam cryostage and annealed at -5, -10, -20, and -24 ºC for 30 min. Optical images were obtained immediately after wafer formation and after an annealing period using an Olympus CX41 microscope equipped with a UIS-2 20x/0.45/∞/0-2/FN22 objective lens, crossed polarisers and a Canon EOS 500D digital camera.

Image analysis was performed in Python using the Segment Anything Model (SAM) to identify ice grain boundaries and segment individual ice crystals [4]. Grain areas were measured from each image, and the mean grain size (MGS) was calculated as the average of three independent images per condition (n=3), with the related error reported as the standard deviation. The IRI activity of DMAA precursor solutions was compared with PEG solutions, which served as a negative control.

Swelling Measurements

Swelling measurements were performed on DMAA cryogels cut into cylindrical specimens (3 mm length × 4.6 mm diameter) immediately after removal from the syringes. The initial mass and diameter of each specimen were recorded as the as-prepared mass (m_0) and as-prepared diameter (D_0). The cryogel specimens were then immersed in 50 mL of ultrapure water at room temperature for 4 days, with the water replaced daily. The mass and diameter were recorded every 24 h until equilibrium was reached, defined as no further change in mass or diameter. At equilibrium, the swollen mass (m_{swl}) and swollen diameter (D_{swl}) were recorded.

Subsequently, the swollen cryogel samples were freeze-dried at -55 ºC and 1 mbar for 72 h. The dry mass (m_{dry}) and dry diameter (D_{dry}) of cryogels were then recorded. Using the measured mass and diameter values, swollen state porosity (P), gel fraction (W_g) and equilibrium water content (EWC) were calculated according to Eq. (S1-S3) given below:

$$P = 1 - q_v . \left(1 + \frac{(q_w - 1)\rho_p}{\rho_{water}}\right)^{-1} \tag{S1}$$

$$W_g = \frac{m_{dry}}{m_0 . C} \tag{S2}$$

$$\text{EWC} = \frac{m_{swollen} - m_{dry}}{m_{swollen}} \tag{S3}$$

where ρ_p and ρ_{water} in Eq. (S1) are densities of DMAA (0.962 g. mL^{-1}), and water (0.997 g.mL^{-1}).

The pore volume (V_p) of DMAA cryogels was determined by swelling freeze-dried samples in acetone, a poor solvent for the DMAA network. Cryogel specimens were immersed in acetone until equilibrium was reached, after which the swollen mass ($m_{swl,\ acetone}$) and swollen diameter ($D_{swl,acet}$) were recorded. V_p was calculated using Eq. (S8).

$$V_p = \left(\frac{m_{swl,acetone}}{m_{dry}} - 1\right). d_{acetone}^{-1} \tag{S4}$$

Rheological Measurements

Rheological measurements were performed using an Anton Paar MCR 702 rheometer equipped with a Peltier temperature control system and a solvent trap to minimise water evaporation at room temperature. Oscillatory strain and frequency sweeps were conducted using a 20 mm diameter parallel-plate geometry. The gap size was set to 1000 ± 200 µm, depending on the thickness of the swollen cryogel specimens.

Cylindrical swollen DMAA cryogel samples were placed between plates, and a preload of 0.02 N was applied to ensure consistent contact before measurements. For strain sweeps, the storage modulus (*G'*), loss modulus (*G''*) and damping factor (*tanδ*) were measured over a strain amplitude range of 0.08-1000 % at a fixed angular frequency (ω) of 10 rad·s^{-1}.

Frequency sweep measurements were performed using the same plate geometry by monitoring *G''*, *G'* and *tanδ* as a function of angular frequency range of 1-100 rad·s^{-1} at a fixed strain amplitude of 0.2%. The linear viscoelastic region (LVR) was determined from strain sweeps and ranged from 0.2 to 1% for all DMAA cryogels. The reported *G'* values were extracted at 0.5% strain within the LVR.

Morphological Analysis with Scanning Electron Microscopy (SEM)

The interior structure and pore morphology of DMAA cryogels were examined using scanning electron microscopy (SEM). Imaging was performed on a FEI Quanta 250 field-emission SEM (Thermo Fisher Scientific) operated at 5 kV. Images were acquired at indicated magnifications.

Before imaging, freeze-dried cryogel samples were sputter-coated with a 10 nm gold-palladium (Au: Pd = 80:20) layer using a Quorum Q150T ES sputter coater (Quorum Technologies Ltd., UK) to ensure electrical conductivity. Pore diameters were quantified using ImageJ software. For each condition, three independent SEM images were analysed, with 100 pores measured per image. Each pore was measured as its equivalent circular diameter (the diameter of a circle with the same cross-sectional area as the pore), which reports the pore width independently of the orientation in which the fracture plane sections the pore. Values are reported as mean ± standard deviation (SD).

Mechanical Testing

Uniaxial compression tests were performed at room temperature using an ElectroForce 5500 universal testing machine (TA Instruments) equipped with a 20 N load cell. Swollen cylindrical DMAA cryogels (3 mm length × 4.6 mm diameter) were used for compression-to-failure, cyclic compression, and fatigue tests.

For compression-to-failure tests, cryogels were compressed to a maximum strain of 95% at a constant crosshead speed of 0.1 mm. s^{-1}. Load (N) versus displacement (mm) was continuously recorded. Engineering strain (ε) and engineering stress (σ) were calculated using the initial specimen length and cross-sectional area.The Young's modulus (*E)* was determined from the slope of the stress-strain curve in the 5-15% strain range. The fracture stress (σ_f) and strain (ε_f) were defined as the maximum stress and corresponding strain before mechanical failure. The toughness (*W*), representing the total energy absorbed before fracture, was calculated as the area under the stress-strain curve. In addition to the cyclic compression tests, fatigue tests were performed by applying 100 successive loading-unloading cycles to cryogels at a maximum strain of up to 60% to evaluate their mechanical stability under repetitive loading.

Differential Scanning Calorimetry (DSC) Measurements

Differential scanning calorimetry (DSC) measurements were performed using a DSC 8000 instrument (PerkinElmer) under a nitrogen atmosphere. DSC was used to investigate the effect of PVA concentration on unfrozen domains in DMAA cryogel precursor solutions. DMAA precursor solutions were prepared as described in the experimental section, except that the initiator and catalyst (APS and TEMED) were not added to prevent polymerisation.

10 mg of each DMAA precursor solution (containing 50 mg. mL^{-1} DMAA monomer, varying PVA concentrations and BAAm crosslinker) was weighed and sealed in aluminium DSC pans. The sample pans were pre-frozen at -20 ºC overnight to ensure complete freezing prior to analysis. The samples were then loaded into the DSC instrument, equilibrated at -20 for 30 min, and heated from -20 to 15 ºC at a rate of 1 ºC.min^{-1}.

The melting enthalpy (ΔH) was determined from the area under the endothermic melting peak, and the melting temperature (T_m) was taken as the peak temperature of the heating curve. The fraction of unfrozen water domains (f_{unf}) was calculated as given in the Eq. (S12)

$$f_{unf} = 1 - \frac{\Delta H}{\Delta Hice} \qquad (S5)$$

where ΔH_{ice} is melting enthalpy of ice which is 333.9 J.g^{-1}

To quantify the cryoconcentration effect, the true concentration of DMAA monomer in the unfrozen domains (C_{true}) was calculated from Eq.(S13) (Supporting Information).

$$C_{true} = \left(\frac{C_{nom}}{C_{nom} + (1-C_{nom}) * f_{unf}}\right) x\ 100 \qquad (S6)$$

Cumulative Dye Release Studies

Dye-release studies of DMAA cryogels were conducted using methylene blue (MB) as a model dye. Water-swollen DMAA cryogels were immersed in 10 mL of 20 µM MB solution prepared in ultrapure water and incubated overnight on an orbital shaker (Ohaus Valour) at 180 rpm to allow dye uptake. The dye-loaded cryogels were then transferred into 10 mL of fresh ultrapure water to initiate dye release and stirred under the same conditions.

At predetermined time points (0, 5, 15, 30, 60, 90, 120, 180, 240, 360, 720, and 1080 min), 100 µL aliquots were withdrawn from the release medium and transferred to a 96-well plate

(Greiner). UV-Vis absorbance measurements were performed using a microplate reader (BioTek Synergy H1) at 660 nm.

Calibration curves were constructed using MB solutions with concentrations ranging from 0.5 to 40 µM, prepared in ultrapure water. The concentration of released MB was determined from the measured absorbance values using the calibration curve.

Column Flow Studies

Column-flow experiments using water-swollen DMAA cryogels (1.5 mm length x 14.5 mm diameter) were performed. A volume of 5 mL of either 20 µM methylene blue (MB) solution or 20 µM mCherry fluorescent protein solution was added to the top of each cryogel column. The breakthrough time, defined as the time required for the first drop to emerge from the column outlet, was recorded in seconds for DMAA cryogels containing 0-5 mg. mL^{-1} PVA.

After completing the passage of MB or mCherry through the cryogel columns, the columns were washed sequentially with phosphate-buffered saline (PBS, pH 7.4) followed by ultrapure water until no further dye or protein was detected in the effluent.

Size-selective separation was evaluated using water-swollen $DMAA_0$ and $DMAA_5$ cryogel columns. A 1:1 mixture of methylene blue (320 Da) and recombinant mCherry (27 kDa) was prepared in milliQ water at 20 µM. Then, 0.4 mL of this mixture was loaded onto each column. Eluted fractions were collected sequentially (0.2 mL per fraction, and 12 fractions were collected). Methylene blue was quantified by absorbance at 660 nm and mCherry by fluorescence (excitation 587 nm/emission 610 nm), both measured on a BioTek Synergy H1 microplate reader in a 96-well plate (Greiner). Concentrations were determined from standards prepared under identical conditions. All measurements were performed at room temperature (n = 3 independent columns).

Fourier Transform Infrared Spectroscopy (FTIR)

To investigate chemical structure of DMAA cryogels, Fourier Transform Infrared Spectroscopy (FTIR, Agilent Technologies Cary 630 FTIR Spectrometer) analysis was done using freeze-dried cryogel samples.

Morphological Analysis with Fluorescence Microscopy

Fluorescence imaging was performed to visualise the internal pore structure of the cryogels. Before imaging, cryogels were left to fully swell in an aqueous solution of fluorescein isothiocyanate (FITC) (0.05 mg mL^{-1}) at room temperature for 12 h in the dark to ensure

homogeneous dye diffusion throughout the gel network. After swelling, excess surface dye was removed by gently rinsing the samples with ultrapure water. Afterwards, the swollen cryogels were sectioned to approximately 200 μm thick to minimise light scattering and enable reliable confocal optical sectioning. The stained cryogels were placed on glass microscope slides and imaged without further chemical treatment. Fluorescence images were obtained using a spinning-disk confocal microscope (Olympus) equipped with appropriate filter sets for FITC detection (excitation/emission: 488/520 nm). All imaging parameters were kept constant across samples.

Turbidity Measurements with UV-Vis Spectroscopy

Turbidity analysis of the DMAA precursor solutions was conducted using an Agilent Cary UV-Vis spectrophotometer equipped with a compact Peltier temperature controller. Measurements were performed on precursor solutions across the full PVA series (0, 1, 2.5, 5 and 10 $mg.mL^{-1}$) without adding the APS/TEMED initiator system. Absorbance was recorded at 400 nm at 25 °C, with an appropriate blank solvent used as a reference. The absence of an increase in absorbance with PVA concentration indicated that no aggregation or precipitation occurred in solution.

Dynamic Light Scattering (DLS)

The hydrodynamic size distributions of species in the DMAA precursor solutions were measured using an Anton Paar Litesizer DLS 700 particle analyser. Measurements were performed on precursor solutions across the full PVA series (0, 1, 2.5, 5 and 10 mg. mL^{-1}) without the APS/TEMED initiator system, at 25 °C. Intensity-weighted size distributions were recorded, and the absence of a micron-scale population at any PVA concentration was taken to confirm that PVA did not aggregate in solution.

S3. Supplementary Figures

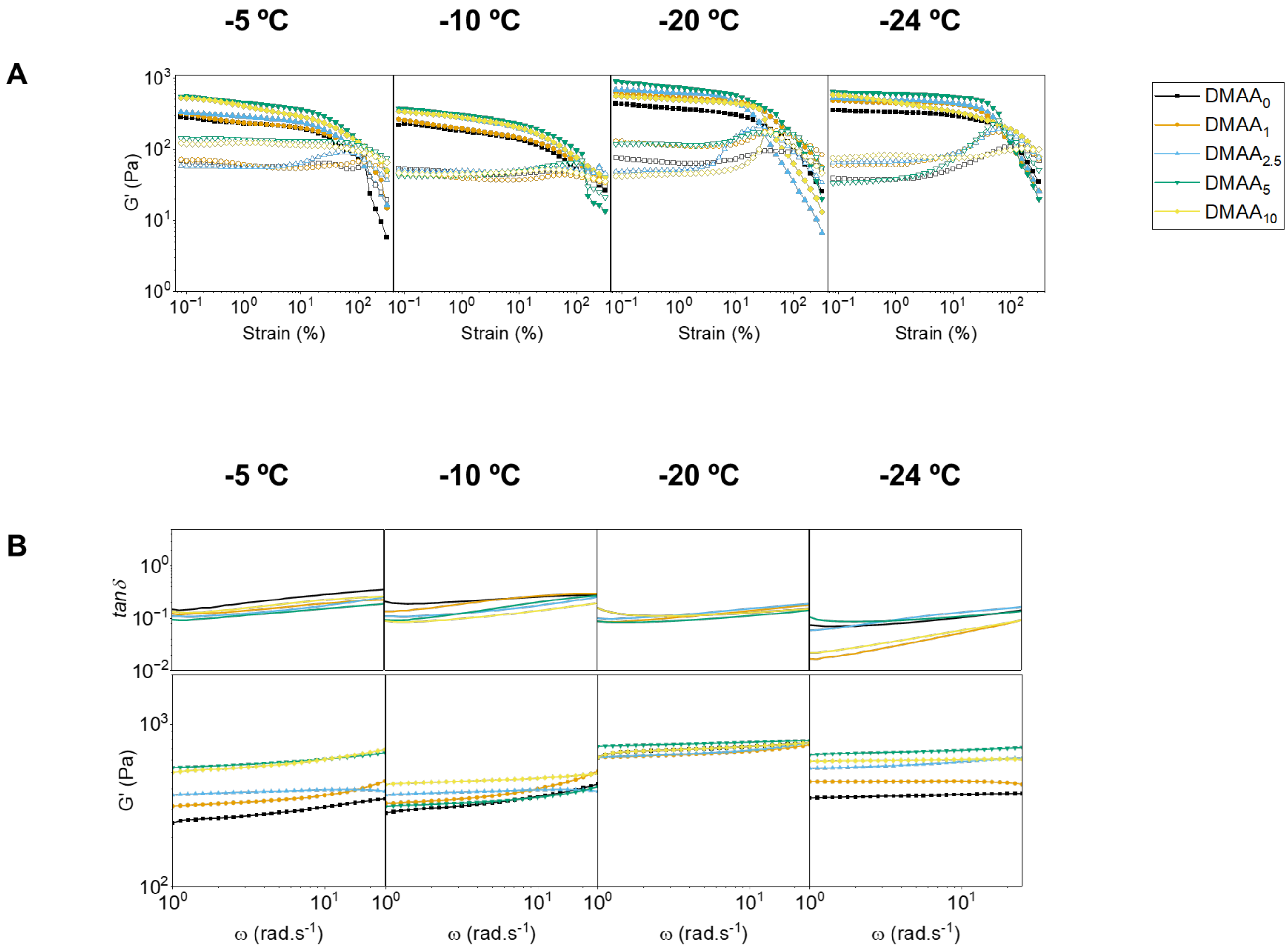

Figure S1. Rheological properties of DMAA cryogels prepared at different PVA concentrations and temperatures. A) Strain sweeps of cryogels (between 0.8 and 100 % strain) versus loss (*G''*, open symbols) and elastic (*G'*, full symbols) modulus at -5, -10, -20 and -24 ºC. B) Frequency sweep of cryogels between 1 and 50 rad. s^{-1} angular frequency (ω) at -5, -10, -20 and -24 ºC.

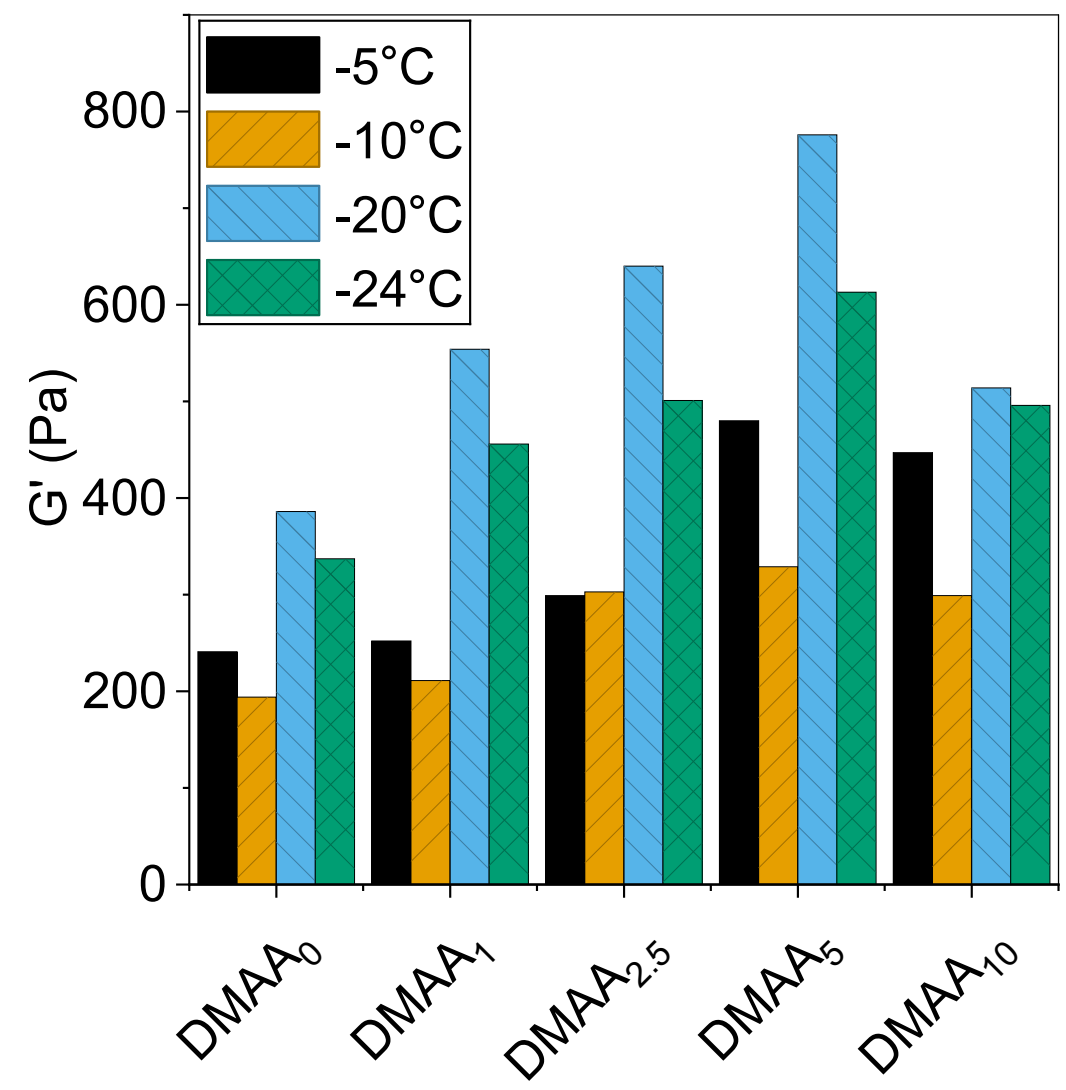

Figure S2. Elastic (*G'*) modulus of DMAA cryogels at -5, -10, -20 and -24 ºC.

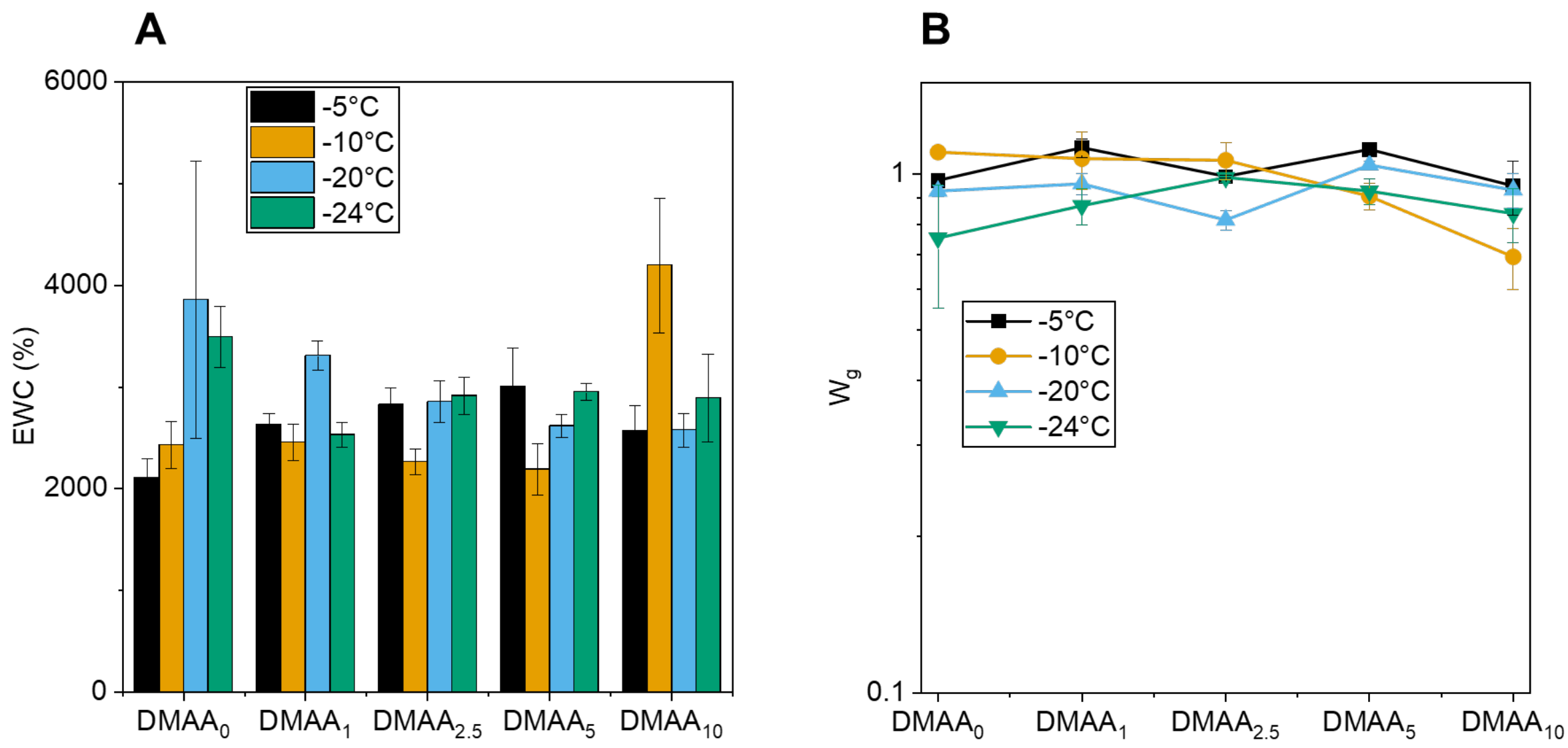

Figure S3. Equilibrium weight swelling ratio (EWC) and gel fraction (W_g) of DMAA cryogels prepared at -5, -10, -20 and -24 ºC as a function of PVA concentration.

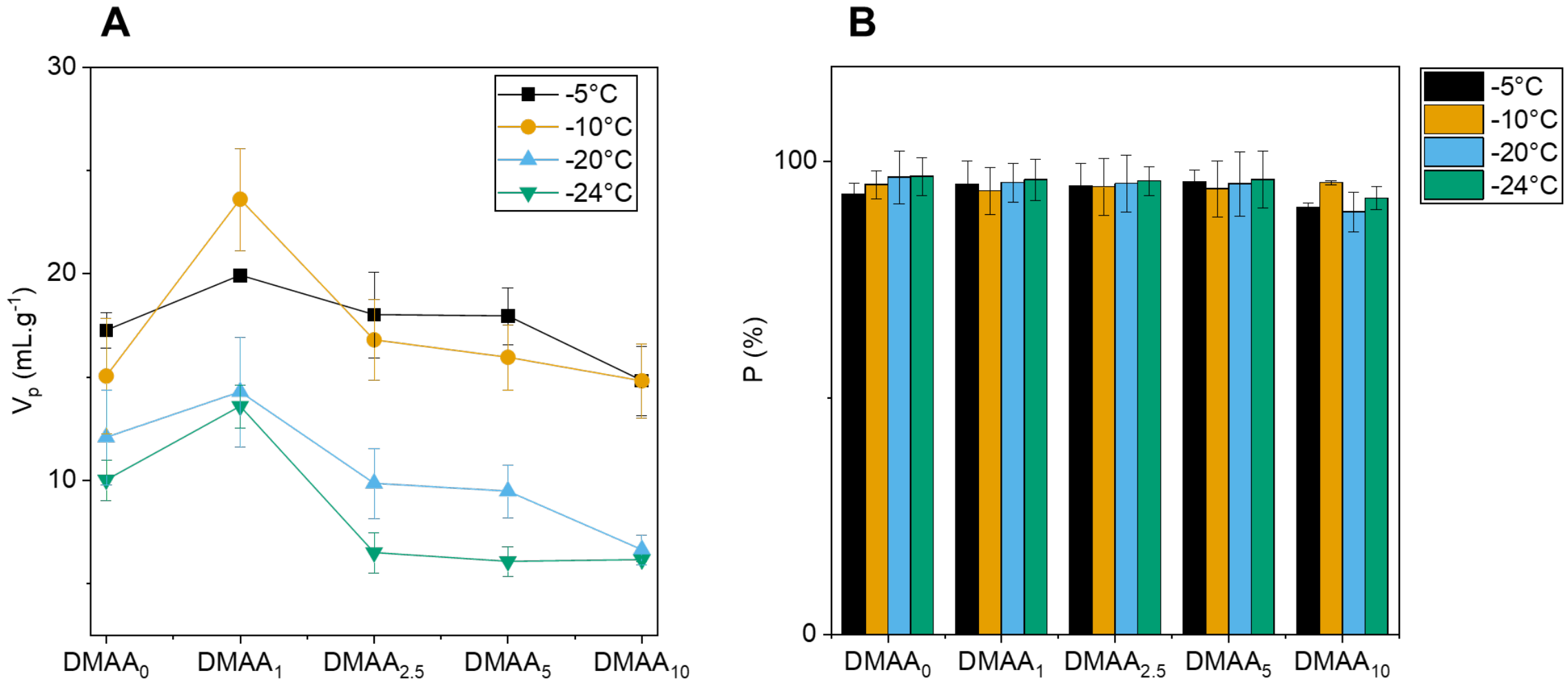

Figure S4. Pore volume (V_p) and swollen state porosity (P) of DMAA cryogels prepared at -5, -10, -20 and -24 ºC.

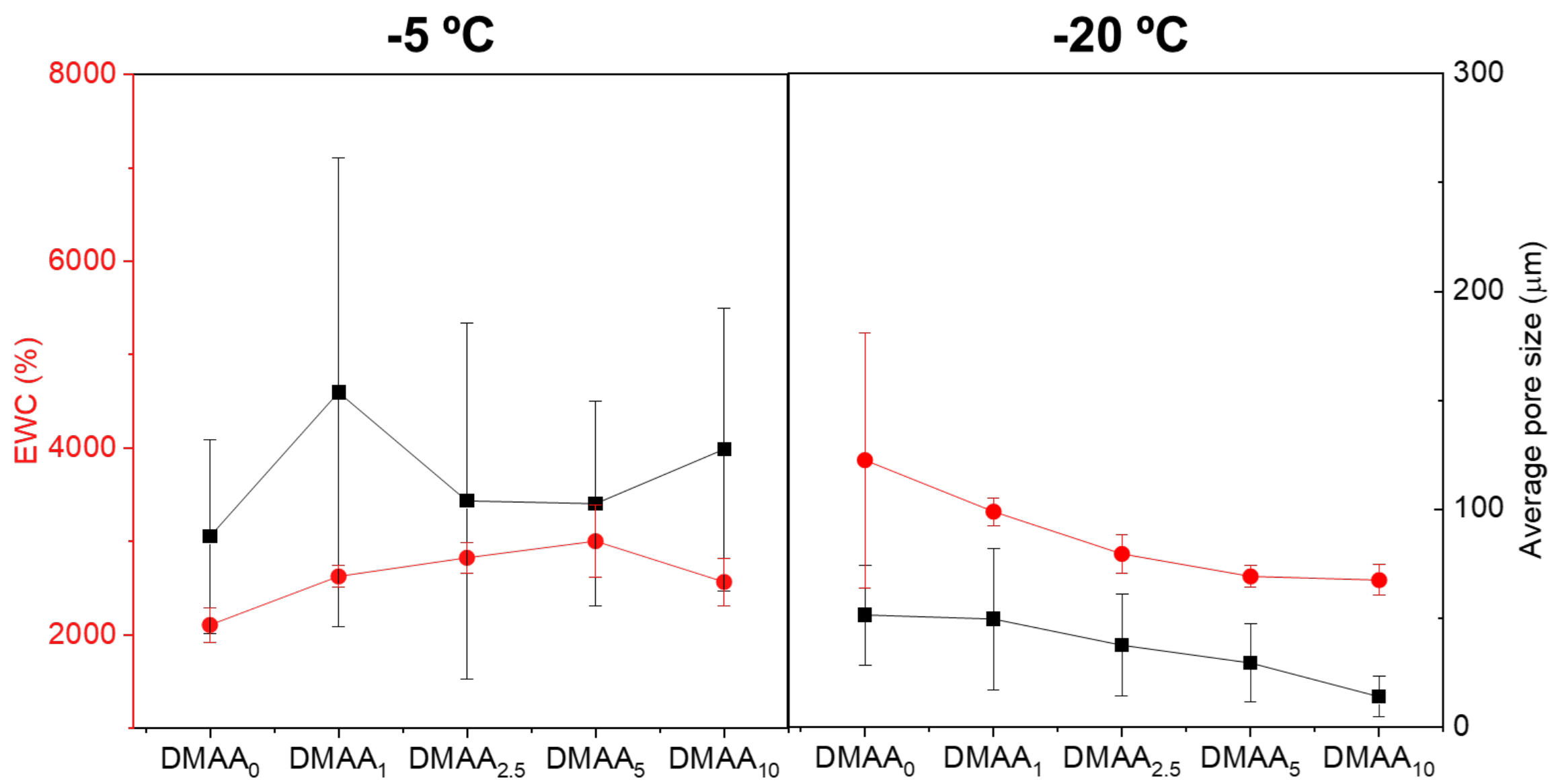

Figure S5. Equilibrium weight swelling ratio (EWC) and average pore size (determined from SEM) of DMAA cryogels prepared at -5, and -20 ºC.

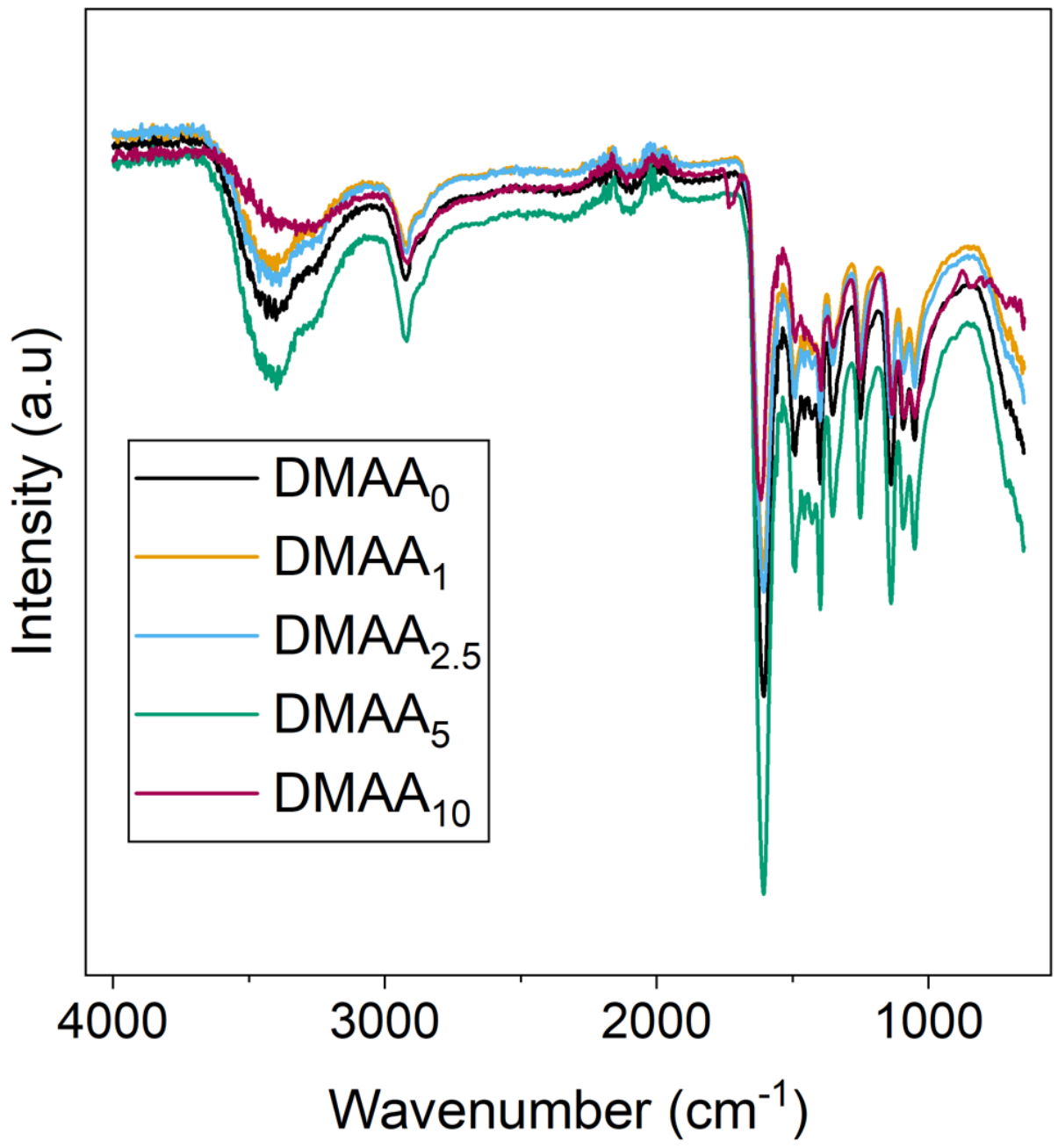

Figure S6. Fourier Transform Infrared spectroscopy **(**FTIR) spectra of DMAA cryogels

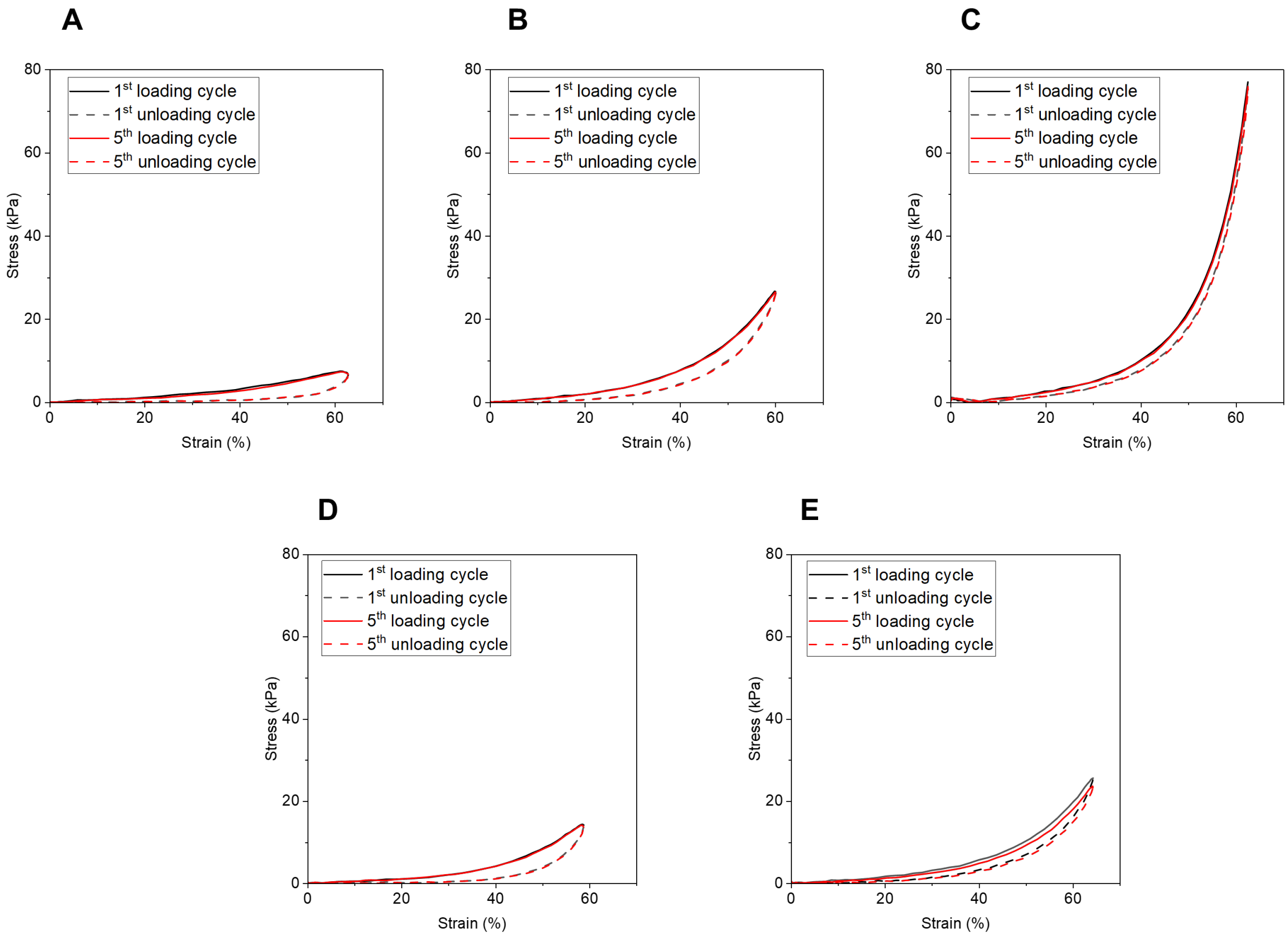

Figure S7. 1st and 5th loading and unloading cycles of 5-cycle long cyclic compression tests of A) $DMAA_0$, B) $DMAA_1$, C) $DMAA_{2.5}$, D) $DMAA_5$, E) $DMAA_{10}$ synthesised at -20 ºC.

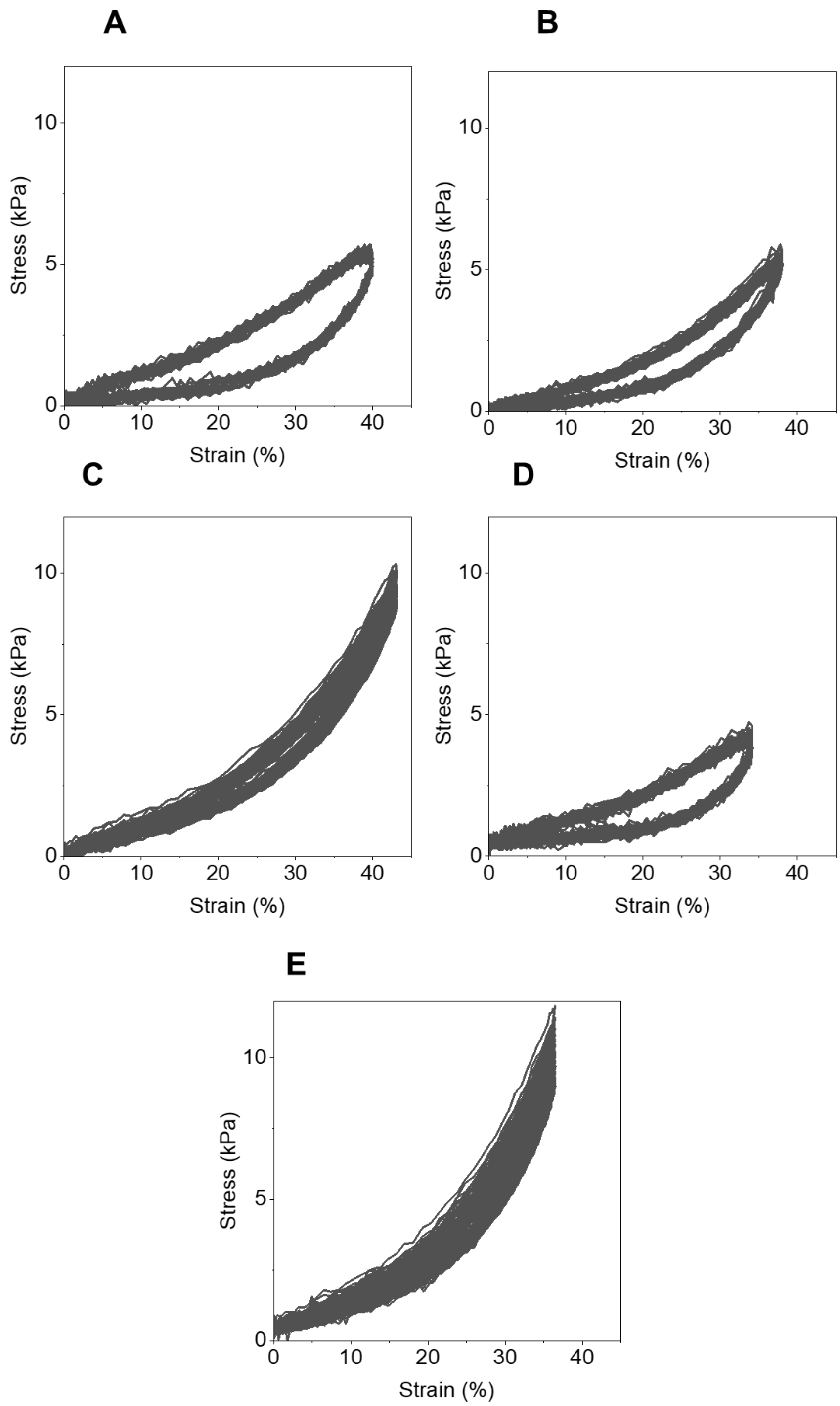

Figure S8. Fatigue resistance tests of A) $DMAA_0$, B) $DMAA_1$, C) $DMAA_{2.5}$, D) $DMAA_5$ and E) $DMAA_{10}$ cryogels under 100 consecutive loading and unloading cycles.

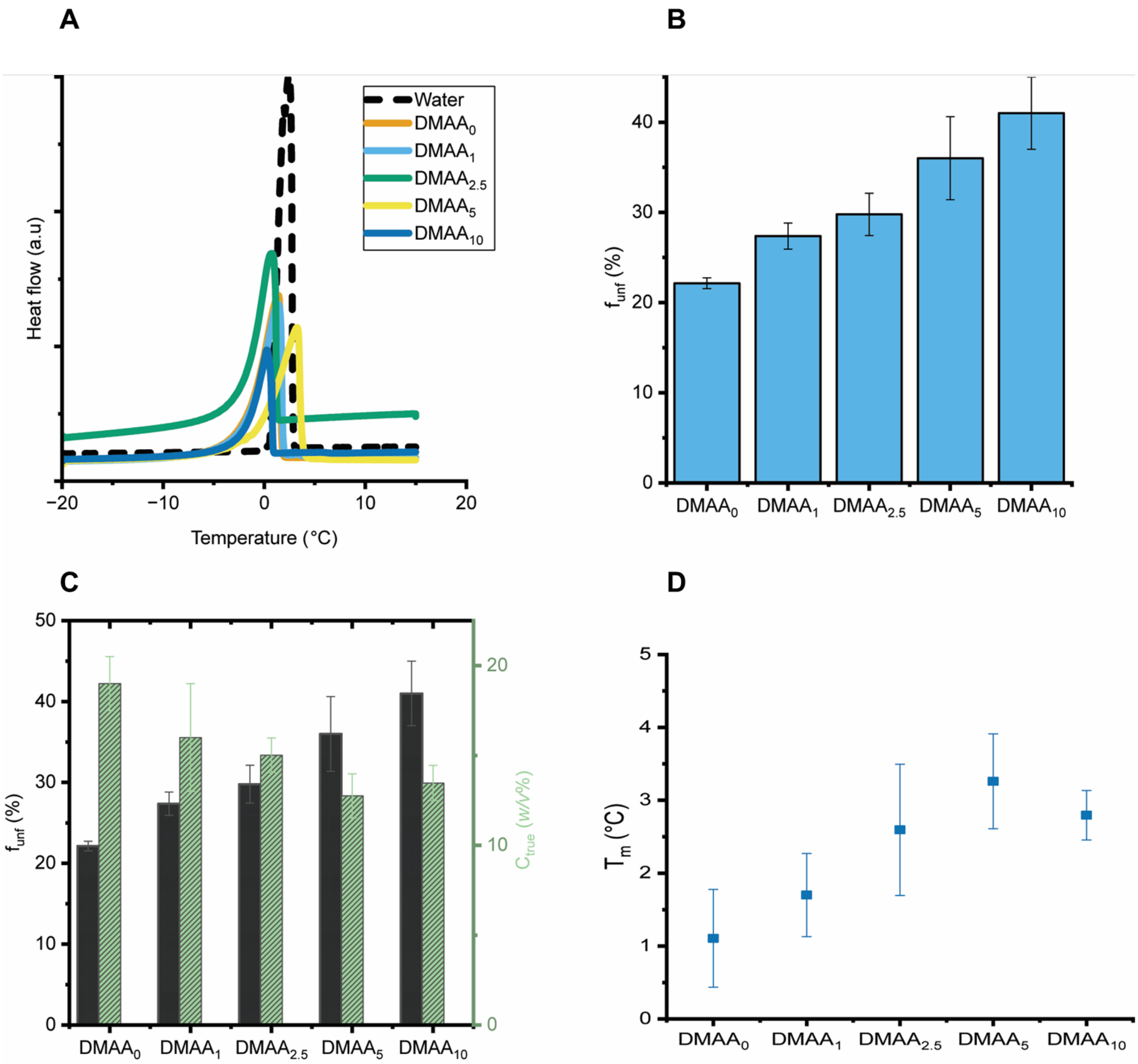

Figure S9. Differential scanning calorimetry (DSC) measurements of DMAA cryogel solutions without initiator system (APS-TEMED). A) Heating curves of DMAA cryogel solutions and water as reference. B) Variations of fraction of unfrozen domains (f_{unf}) of DMAA cryogel solutions. C) f_{unf} and true concentration of monomers in the unfrozen domains of DMAA cryogel solutions. D) Melting temperature of DMAA cryogel solutions that obtained from DSC scans.

A B C D

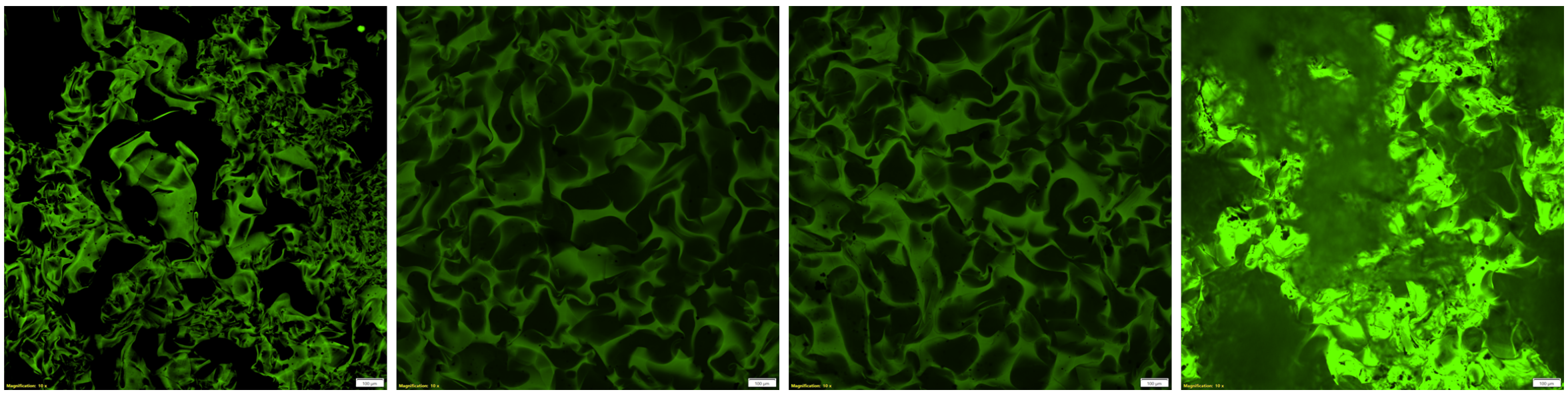

Figure S10. Confocal fluorescence microscopy images of water-swollen A) $DMAA_0$ B) $DMAA_{2.5}$ C) $DMAA_5$ and D) $DMAA_{10}$ cryogels. Scale bars: 100 µm.

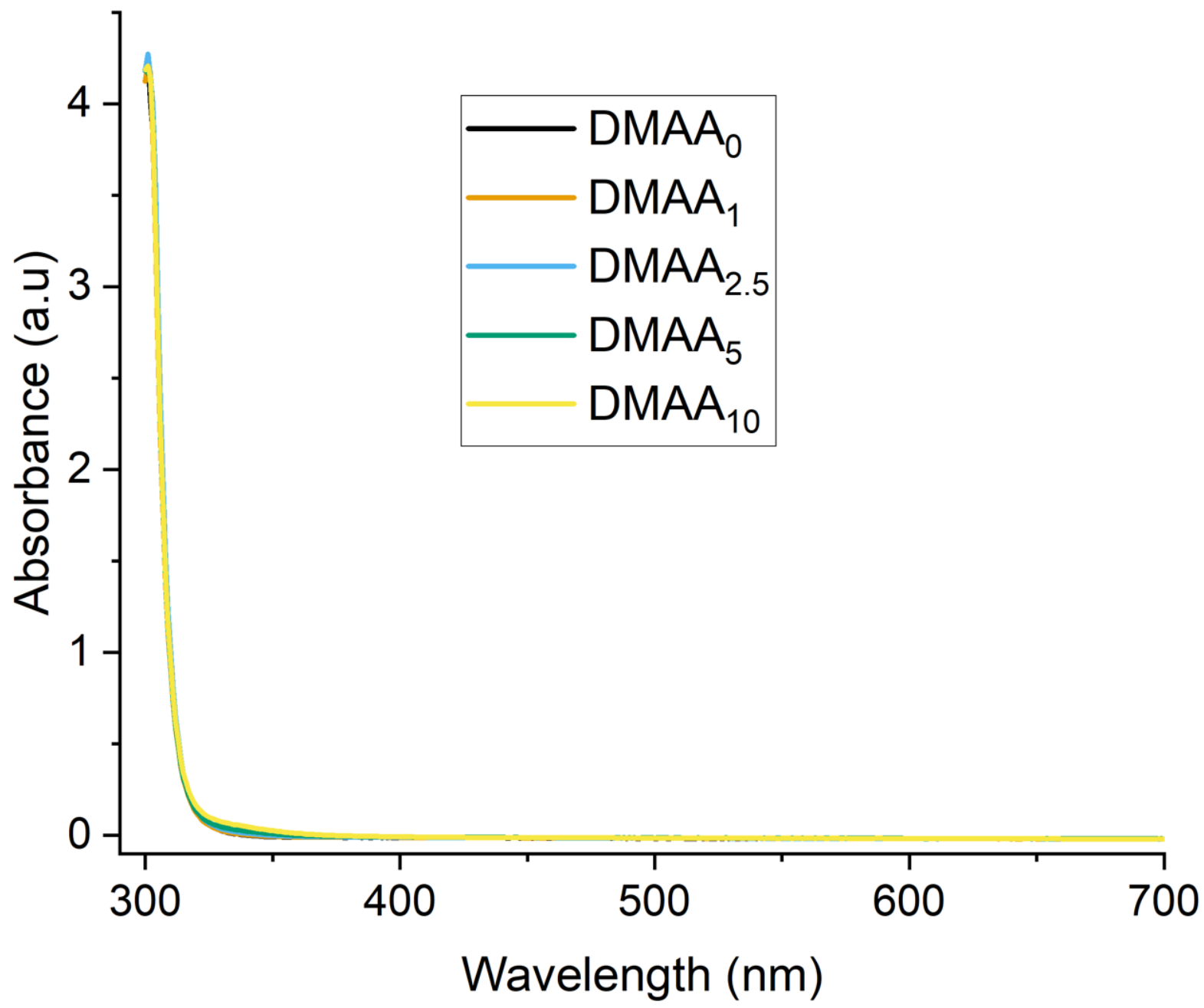

Figure S11. UV-vis turbidity of DMAA precursor solutions across the full PVA series (0, 1, 2.5, 5 and 10 mg. mL^{-1}).

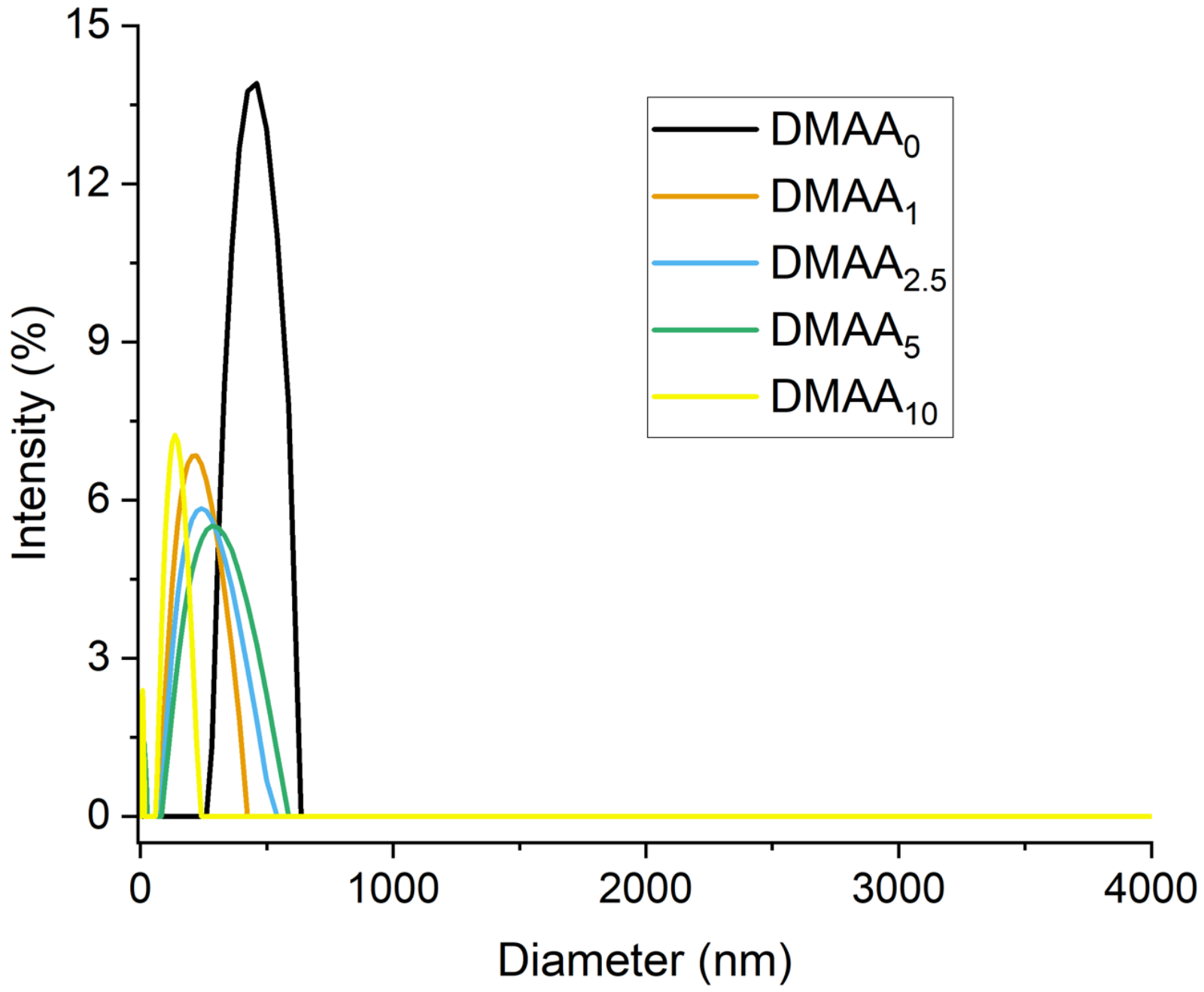

Figure S12. Dynamic light scattering (DLS) intensity distributions of DMAA precursor solutions across the full PVA series (0, 1, 2.5, 5 and 10 mg. mL^{-1}).

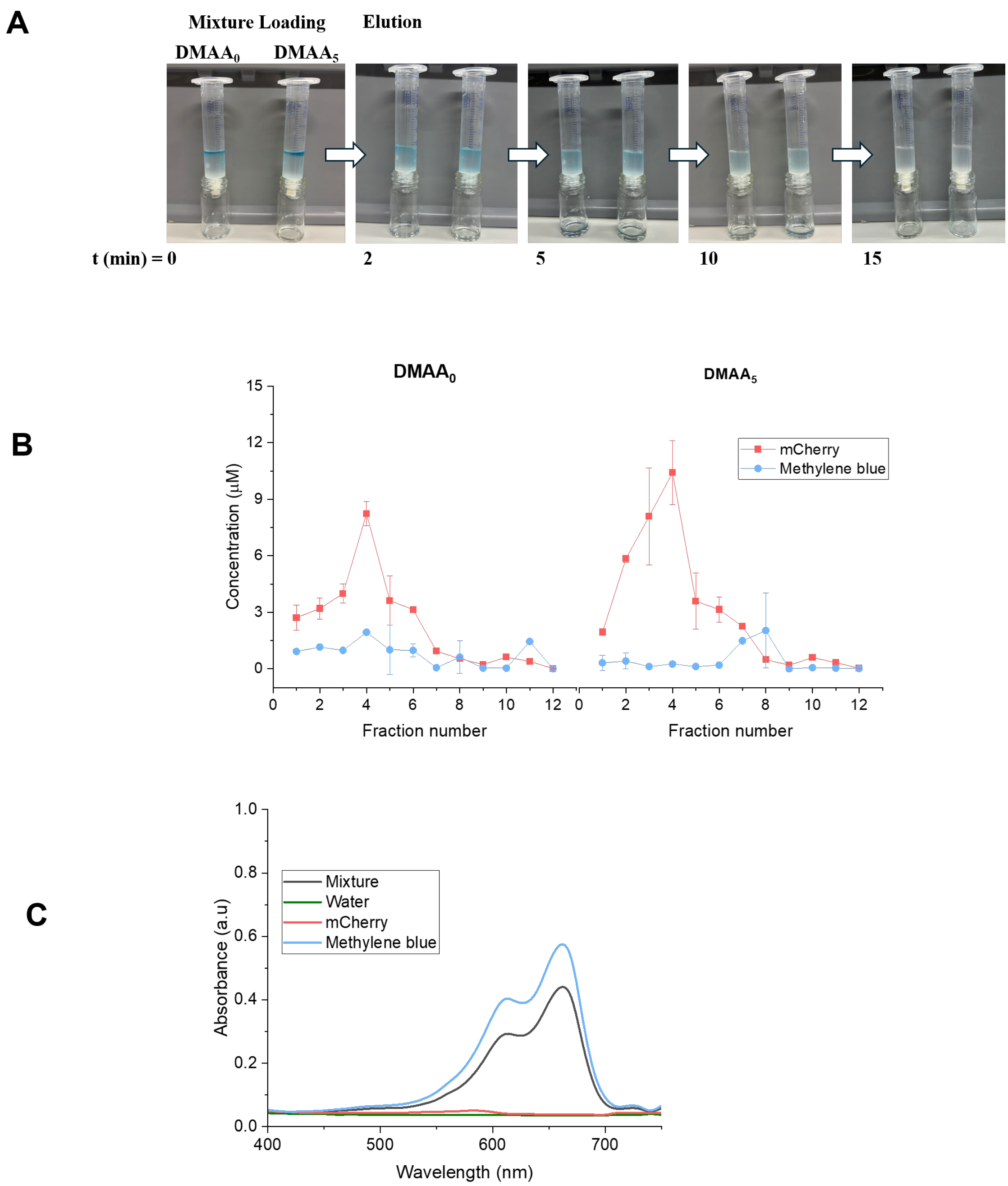

Figure S13. Pore size-dependent separation of a methylene blue and mCherry mixture on pore-programmed DMAA cryogel columns. **(A)** 1:1 mixture of methylene blue (320 Da) and mCherry (27 kDa), each 20 µM, loaded and eluted through $DMAA_0$ (no PVA, large pore) and $DMAA_5$ (5 mg.mL^{-1} PVA, small pore) columns. The visible blue band is methylene blue, while mCherry is not visible to the eye at this concentration and is tracked by fluorescence. **(B)** Elution profiles showing the concentration of mCherry (fluorescence, excitation 587 / emission 610 nm) and methylene blue (absorbance, 660 nm), in each fraction in µM. (mean ± standard deviation, n = 3 independent cryogel columns). **(C)** UV-vis spectra of the mixture and standards.

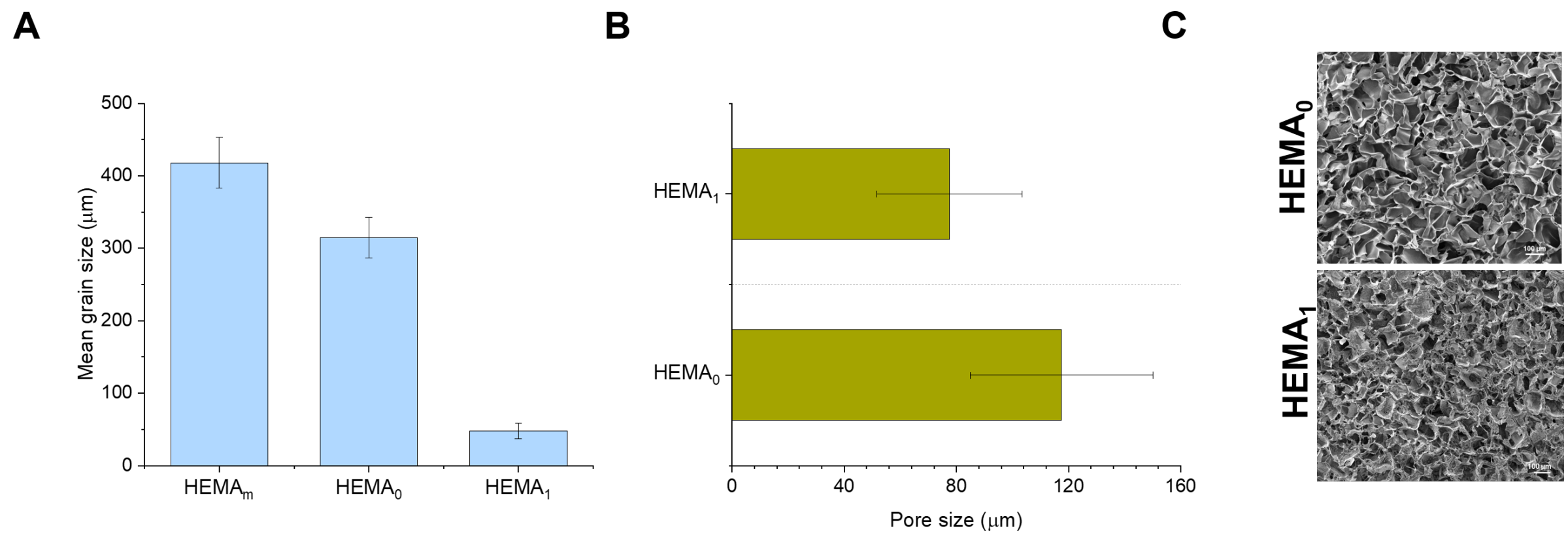

Figure S14. Generalisation of the ICE-CAP approach to a second cryogel system (pHEMA). **(A)** Mean ice grain size from splat assays of HEMA precursor solutions. ($HEMA_{m,}$ $HEMA_0$, $HEMA_1$, $HEMA_{10)}$. **(B)** Pore size of $HEMA_0$ and $HEMA_1$ cryogels. (C) Corresponding SEM images of $HEMA_0$ and $HEMA_1$ cryogels. Scale bars = 100 μm. $HEMA_m$ = monomer-only control, $HEMA_{0/1}$ = 0 and 1 PVA).

S4. Supplementary Tables

Table S1. Comparison of pore size, elastic modulus, f_{unf}, C_{true} and T_m of cryogels across different PVA concentrations.

Sample	Pore size (µm)	Elastic modulus (Pa)	f_{unf} (%)	C_{true} (%)	T_m (ºC)
**$DMAA_0$**	51.5 ± 22.9	386 ± 42	22.1 ± 0.6	19.0 ± 1.5	1.11 ± 0.67
**$DMAA_1$**	49.6 ± 32.5	554 ± 14	27.4 ± 1.4	16.0 ± 3.0	1.70 ± 0.57
**$DMAA_{2.5}$**	37.6 ± 23.3	640 ± 32	29.8 ± 2.3	15.0 ± 1.0	2.60 ± 0.90
**$DMAA_5$**	29.5 ± 17.8	776 ± 56	36.0 ± 4.6	12.8 ± 1.2	3.26 ± 0.65
**$DMAA_{10}$**	14.0 ± 9.3	514 ± 43	41.0 ± 4.0	13.5 ± 1.0	2.80 ± 0.34

S5. Python Code for Image Analysis

The following Python script was used to segment ice crystals from microscope images obtained during splat ice recrystallisation inhibition (IRI) assays. Image segmentation was performed using the Segment Anything Model (SAM). The script outputs individual grain masks, grain areas (in pixels), and grain size distributions, which were used to calculate the mean grain size (MGS) reported in the main manuscript. (Python version: 3.10, Required packages: torch, segment-anything, numpy, opencv-python, matplotlib, h5py, supervision, Hardware: CPU/GPU agnostic)

```
import tkinter as tk
from tkinter import filedialog, messagebox, scrolledtext
import threading
import os, torch, cv2, h5py, datetime
import numpy as np
from segment_anything import sam_model_registry, SamAutomaticMaskGenerator
import supervision as sv
import matplotlib.pyplot as plt

os.system("cls")

# ---- CONFIG ----
CHECKPOINT_PATH = "sam_vit_h_4b8939.pth"
MODEL_TYPE = "vit_h"
DEVICE = torch.device('cuda:0' if torch.cuda.is_available() else 'cpu')

# ---- LOAD MODEL ----
sam                                                                    =
sam_model_registry[MODEL_TYPE](checkpoint=CHECKPOINT_PATH).to(device=DEVIC
E)
mask_generator = SamAutomaticMaskGenerator(sam)

# ---- MAIN FUNCTION ----
def segment_image(image_path, save_hdf5, save_csv, save_histogram, log_fn):
    try:
        start_t = datetime.datetime.now()
        log_fn(f"Started at: {start_t}")

        image_bgr = cv2.imread(image_path)
        if image_bgr is None:
            log_fn("Error: Could not load image.")
            return
        image_rgb = cv2.cvtColor(image_bgr, cv2.COLOR_BGR2RGB)
        log_fn("Image loaded...")

        sam_result = mask_generator.generate(image_rgb)
        log_fn(f"SAM results loaded: {list(sam_result[0].keys())}")
```

```
        mask_annotator                                                   =
sv.MaskAnnotator(color_lookup=sv.ColorLookup.INDEX)
        detections = sv.Detections.from_sam(sam_result=sam_result)
        annotated_image  =  mask_annotator.annotate(scene=image_bgr.copy(),
detections=detections)

        masks  =  [mask['segmentation']  for  mask  in  sorted(sam_result,
key=lambda x: x['area'], reverse=True)]

        for i, mask in enumerate(masks):
            M = cv2.moments(mask.astype(np.uint8))
            if M["m00"] != 0:
                cX = int(M["m10"] / M["m00"])
                cY = int(M["m01"] / M["m00"])
                cv2.putText(
                    annotated_image,
                    str(i + 1),
                    (cX, cY),
                    cv2.FONT_HERSHEY_SIMPLEX,
                    0.6,
                    (255, 255, 255),
                    2,
                    cv2.LINE_AA
                )

        base_name = os.path.splitext(os.path.basename(image_path))[0]

        if save_hdf5:
            with h5py.File(f"Masks_{base_name}.h5", "w") as file:
                for i, m in enumerate(masks):
                    file.create_dataset(f"{i}", data=m)
            log_fn("HDF5 file saved.")

        areas = np.array([np.count_nonzero(m) for m in masks])

        if save_csv:
            grains_nums = np.arange(1, len(masks)+1).reshape(-1, 1)
            export_data = np.hstack((grains_nums, areas.reshape(-1, 1)))
            np.savetxt(f"GrainAreas_{base_name}.csv",         export_data,
delimiter=",", header="Grain Number,Area px2")
            log_fn("CSV file saved.")

        if save_histogram:
            plt.switch_backend('TkAgg')   #  Switch  backend  for  GUI-safe
plotting
            fig, ax = plt.subplots()
            num_bins = max(1, len(masks)//12)
            ax.set_title(f"Area:                       {np.mean(areas):.2E}
(std={np.std(areas):.2E}) [px^2]")
            ax.set_xlabel("Grain Area [px^2]")
            ax.set_ylabel("Number of Grains")
            ax.hist(areas,         bins=num_bins,         facecolor='red',
edgecolor="black", alpha=0.5)
            fig.savefig(f"Distribution_{base_name}.png")
```

```
            plt.close(fig)
            log_fn("Histogram saved.")

        cv2.imwrite(f"Segmented_{base_name}.png", annotated_image)
        log_fn("Segmented image saved.")
        log_fn(f"Finished in {datetime.datetime.now()-start_t}")

    except Exception as e:
        log_fn(f"Error: {e}")

# ---- GUI ----
def run_gui():
    def browse_file():
        file_path = filedialog.askopenfilename(filetypes=[("Image Files",
"*.jpg *.jpeg *.png")])
        if file_path:
            image_path_var.set(file_path)

    def start_segmentation():
        img_path = image_path_var.get()
        if not img_path:
            messagebox.showerror("Error", "Please select an image file.")
            return
        segment_btn.config(state=tk.DISABLED)

        def run_in_thread():
            segment_image(
                img_path,
                hdf5_var.get(),
                csv_var.get(),
                hist_var.get(),
                log_message
            )
            root.after(0, lambda: segment_btn.config(state=tk.NORMAL))

        threading.Thread(target=run_in_thread, daemon=True).start()

    def log_message(msg):
        log_text.config(state=tk.NORMAL)
        log_text.insert(tk.END, msg + "\n")
        log_text.see(tk.END)
        log_text.config(state=tk.DISABLED)

    root = tk.Tk()
    root.title("SAM Image Segmenter")

    tk.Label(root, text="Select Image:").grid(row=0, column=0, sticky="w",
padx=5, pady=5)
    image_path_var = tk.StringVar()
    tk.Entry(root,   textvariable=image_path_var,   width=50).grid(row=0,
column=1, padx=5, pady=5)
    tk.Button(root,    text="Browse",    command=browse_file).grid(row=0,
column=2, padx=5, pady=5)
```

```
    hdf5_var = tk.BooleanVar(value=False)
    csv_var = tk.BooleanVar(value=True)
    hist_var = tk.BooleanVar(value=True)

    tk.Checkbutton(root, text="Save HDF5", variable=hdf5_var).grid(row=1,
column=0, sticky="w", padx=5)
    tk.Checkbutton(root, text="Save CSV", variable=csv_var).grid(row=1,
column=1, sticky="w", padx=5)
    tk.Checkbutton(root, text="Save Histogram",
variable=hist_var).grid(row=1, column=2, sticky="w", padx=5)

    segment_btn = tk.Button(root, text="Segment",
command=start_segmentation)
    segment_btn.grid(row=2, column=1, pady=10)

    tk.Label(root, text="Log:").grid(row=3, column=0, sticky="nw", padx=5)
    log_text = scrolledtext.ScrolledText(root, height=15, width=70,
state=tk.DISABLED)
    log_text.grid(row=4, column=0, columnspan=3, padx=5, pady=5)

    root.mainloop()

if __name__ == "__main__":
    run_gui()
```

References

1. Stephens, A.D., Lu, M., Fernandez-Villegas, A., and Kaminski Schierle, G.S. (2020) Fast Purification of Recombinant Monomeric Amyloid-β from E. coli and Amyloid-β-mCherry Aggregates from Mammalian Cells. *ACS Chem. Neurosci.*, **11** (20), 3204–3213.
2. Doser, G., Su, E., and Okay, O. (2023) Effects of cryogenic condition and chemistry on the properties of synthetic and biopolymer cryogels. *Reactive and Functional Polymers*, **190**, 105635.
3. Knight, C.A., Hallett, J., and DeVries, A.L. (1988) Solute effects on ice recrystallization: An assessment technique. *Cryobiology*, **25** (1), 55–60.
4. Archit, A., Freckmann, L., Nair, S., Khalid, N., Hilt, P., Rajashekar, V., Freitag, M., Teuber, C., Spitzner, M., Tapia Contreras, C., Buckley, G., von Haaren, S., Gupta, S., Grade, M., Wirth, M., Schneider, G., Dengel, A., Ahmed, S., and Pape, C. (2025) Segment Anything for Microscopy. *Nat Methods*, **22** (3), 579–591.
